# Supplementary material for: Differential Myocardial Responses in Male and Female Rats with Uremic Cardiomyopathy
Source: Int J Mol Sci. 2025 Mar 3;26(5):2259. doi: 10.3390/ijms26052259 (PMC11900185; doi:10.3390/ijms26052259)

Supplementary Material – Full Western Blot Membranes and Gels

Left ventricle_Cardiac Troponin I_original membranes_ Ser-22/23-P

S1A

1. Sham Male
2. CKD Male
3. Sham Female
4. CKD Female

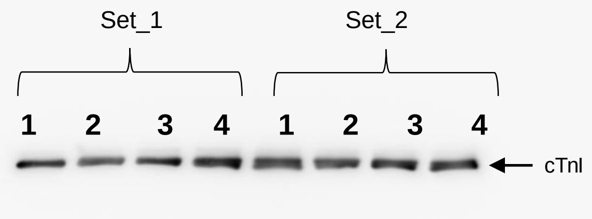

Left ventricle_Cardiac Troponin I_original membranes_ Ser-22/23-P-Blot stain

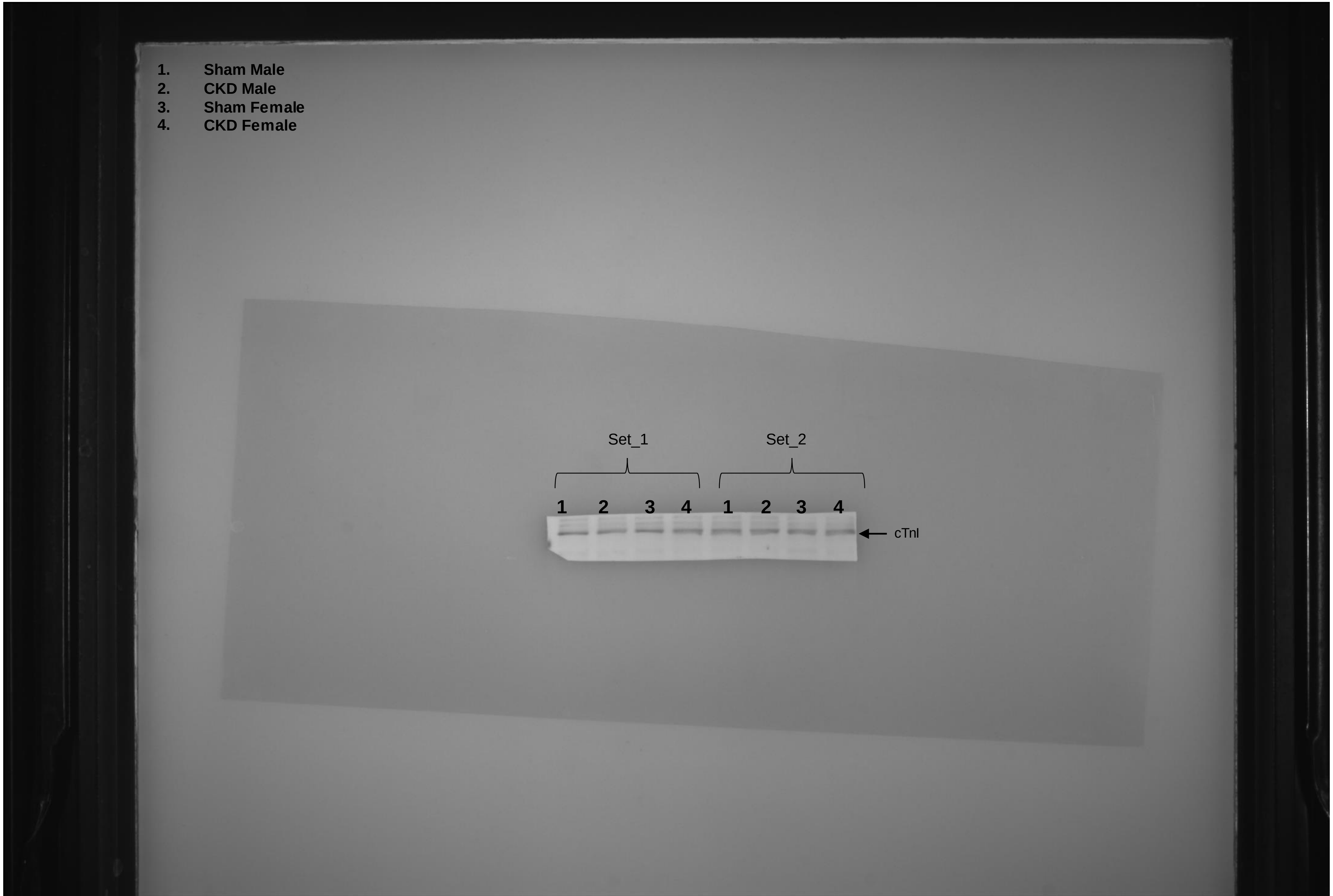

Left ventricle_Cardiac Troponin I_original membranes_ Ser-22/23-P

S2A

1. **Sham Male**
2. **CKD Male**
3. **Sham Female**
4. **CKD Female**

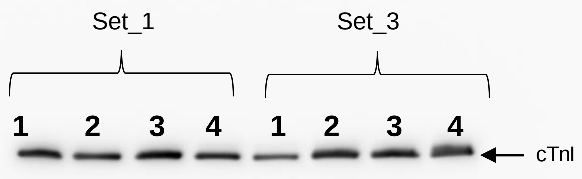

Left ventricle_Cardiac Troponin I_original membranes_ Ser-22/23-P-Blot stain

S2B

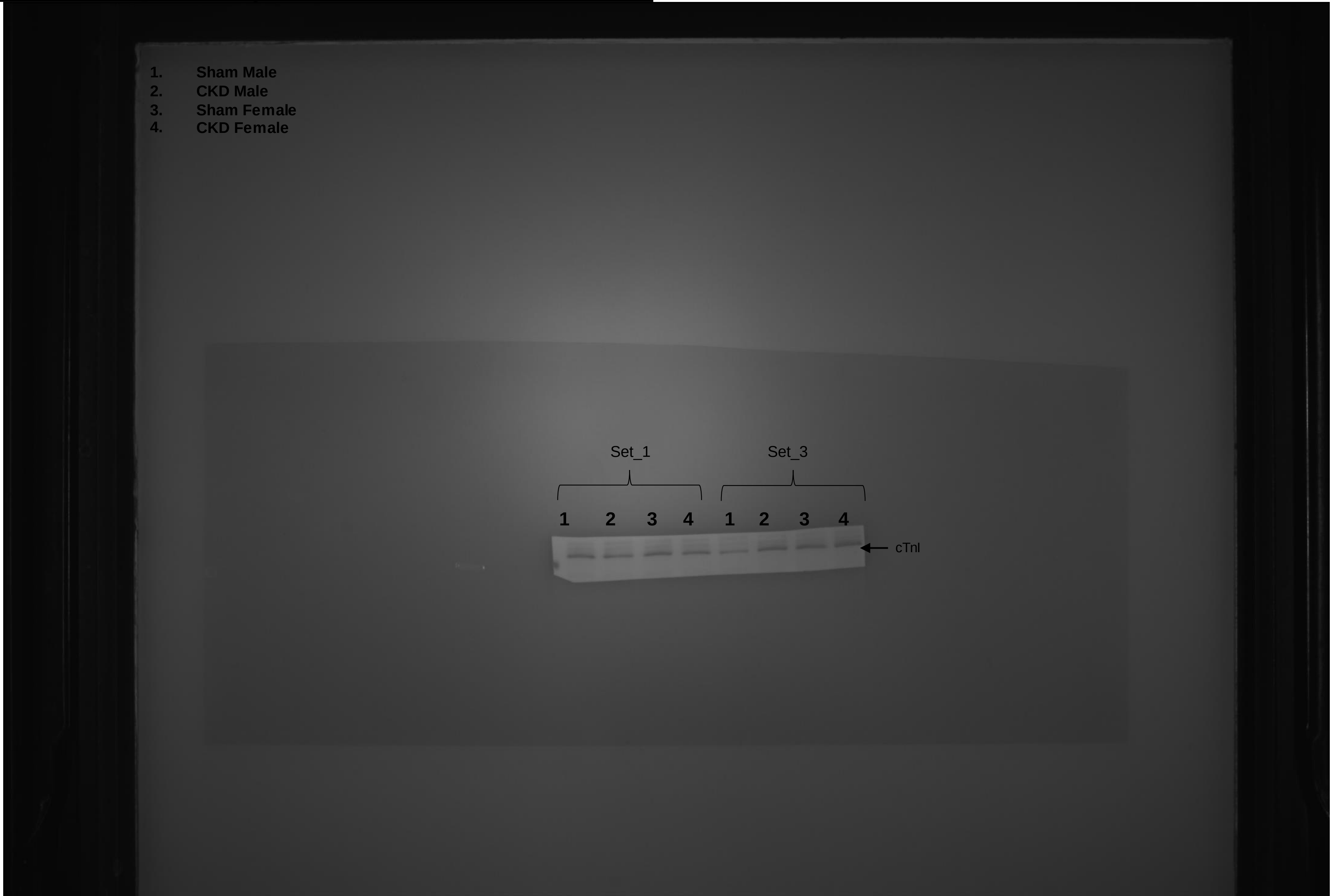

Left ventricle_Cardiac Troponin I_original membranes_ Ser-22/23-P

S3A

1. Sham Male
2. CKD Male
3. Sham Female
4. CKD Female

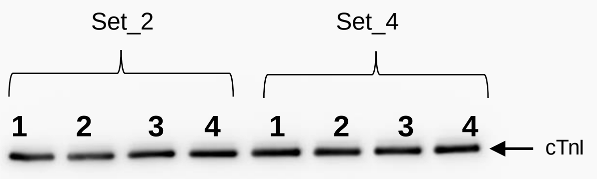

Left ventricle_Cardiac Troponin I_original membranes_ Ser-22/23-P-Blot stain

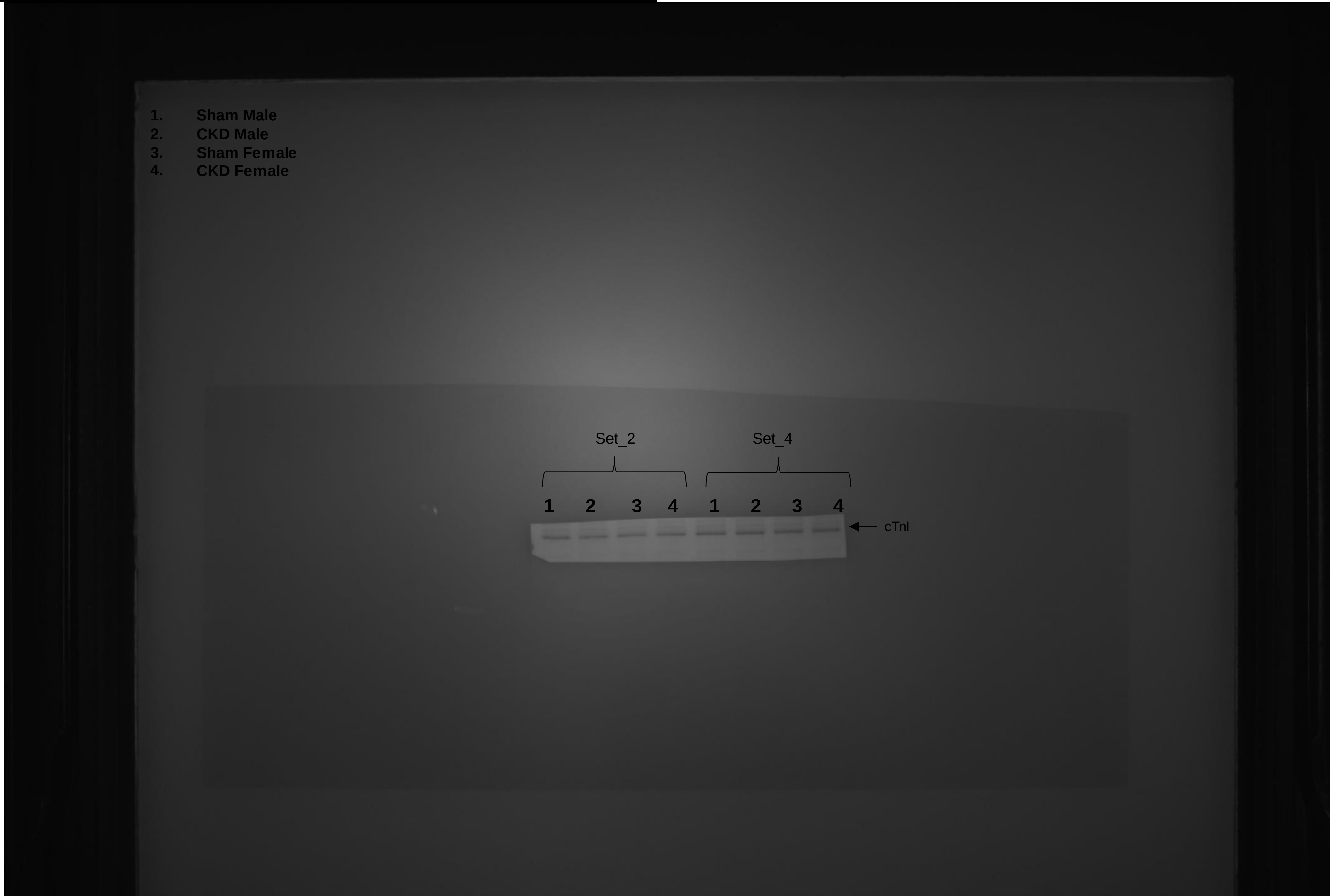

Left ventricle_Cardiac Troponin I_original membranes_ Ser-22/23-P

S4A

1. Sham Male
2. CKD Male
3. Sham Female
4. CKD Female

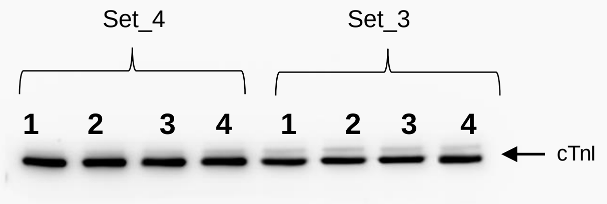

Left ventricle_Cardiac Troponin I_original membranes_ Ser-22/23-P-Blot stain

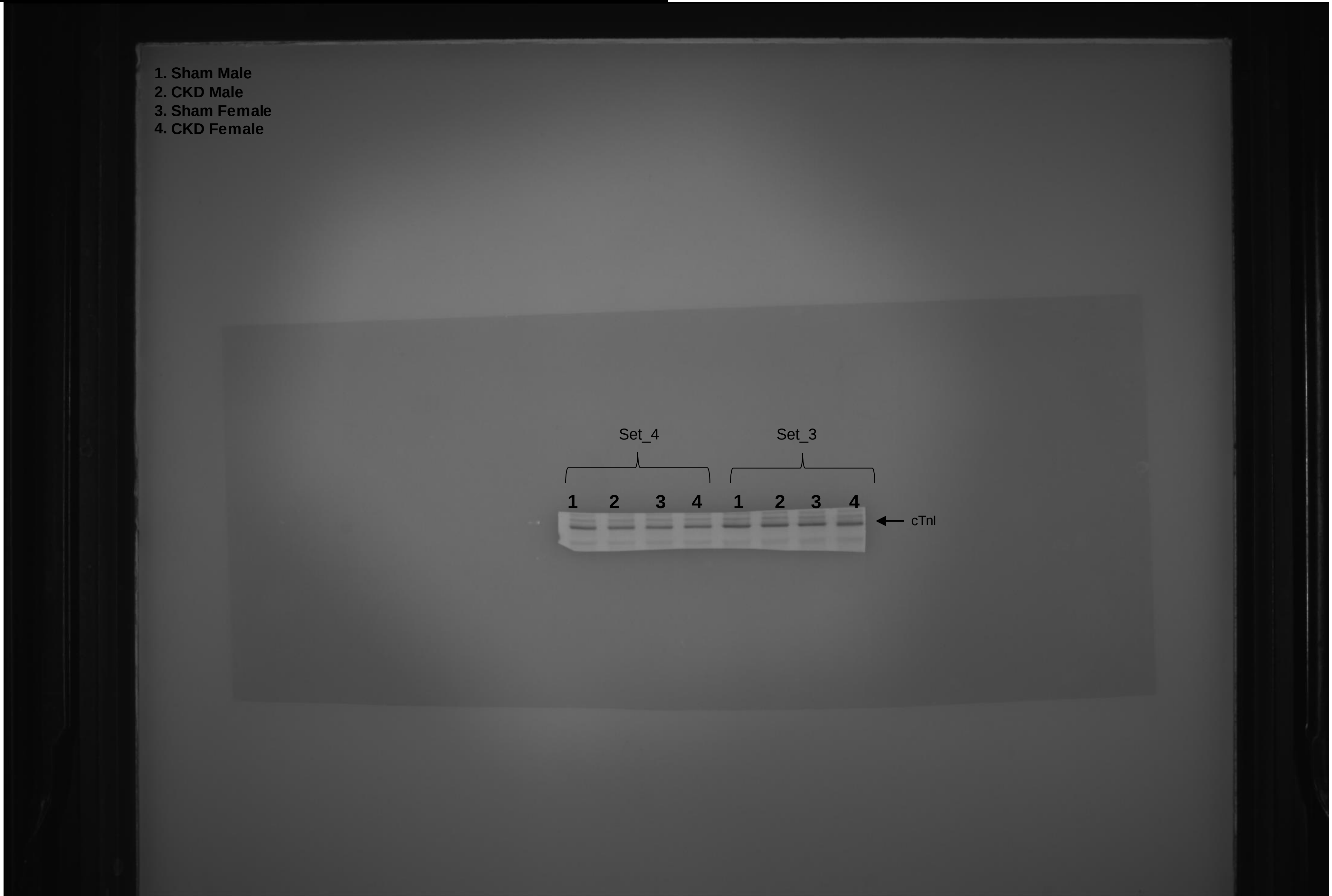

Left ventricle_Cardiac Troponin I_original membranes_ Ser-22/23-P

S5A

1. Sham Male
2. CKD Male
3. Sham Female
4. CKD Female

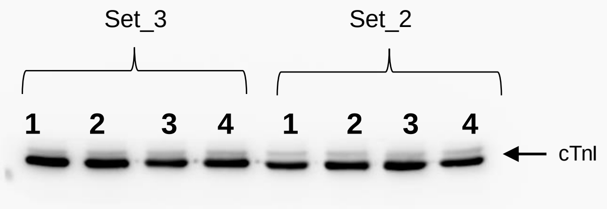

Left ventricle_Cardiac Troponin I_original membranes_ Ser-22/23-P-Blot stain

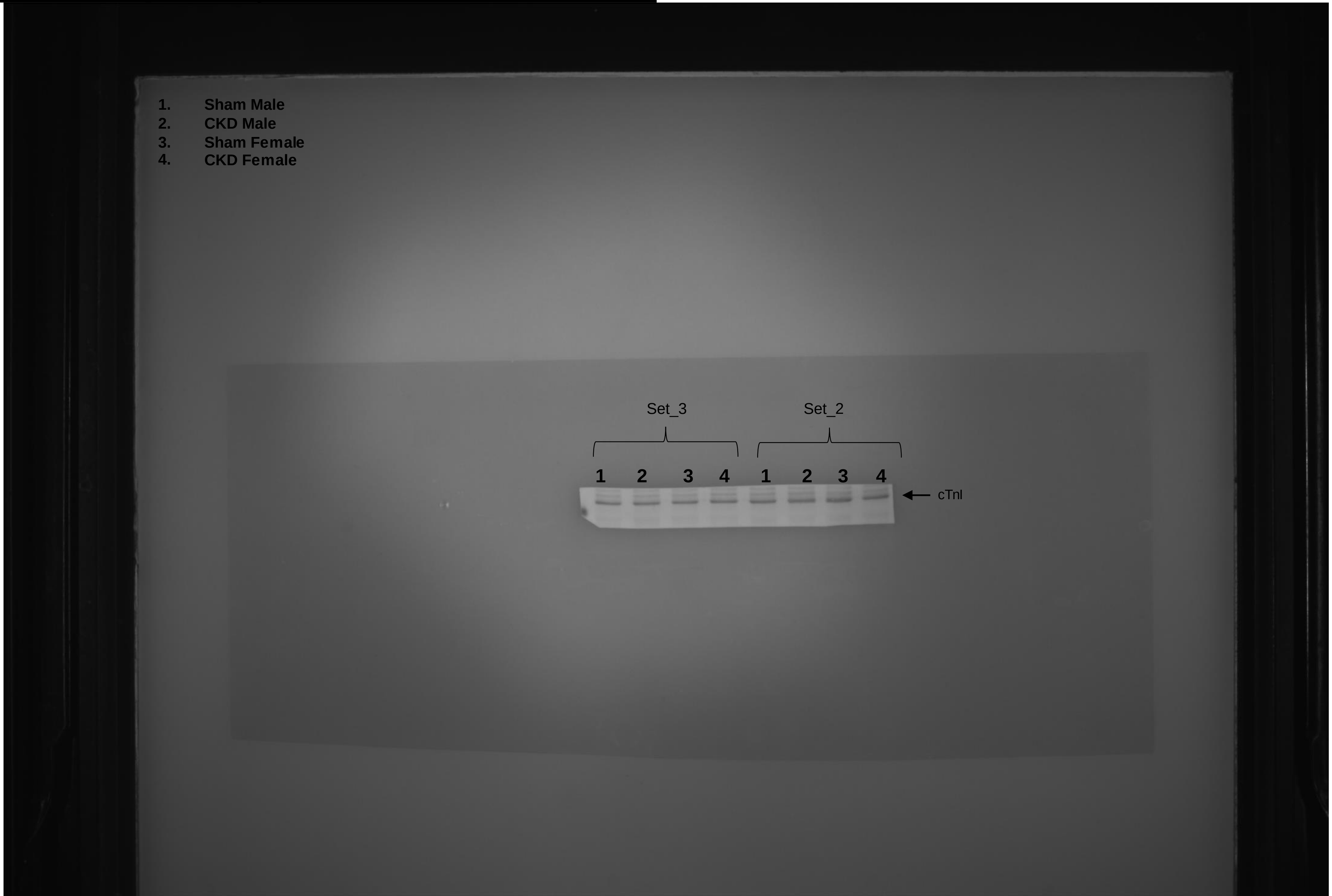

Left ventricle_Cardiac Troponin I_original membranes_ Ser-22/23-P

S6A

1. Sham Male
2. CKD Male
3. Sham Female
4. CKD Female

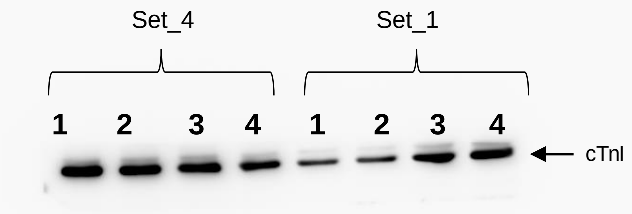

Left ventricle_Cardiac Troponin I_original membranes_ Ser-22/23-P-Blot stain

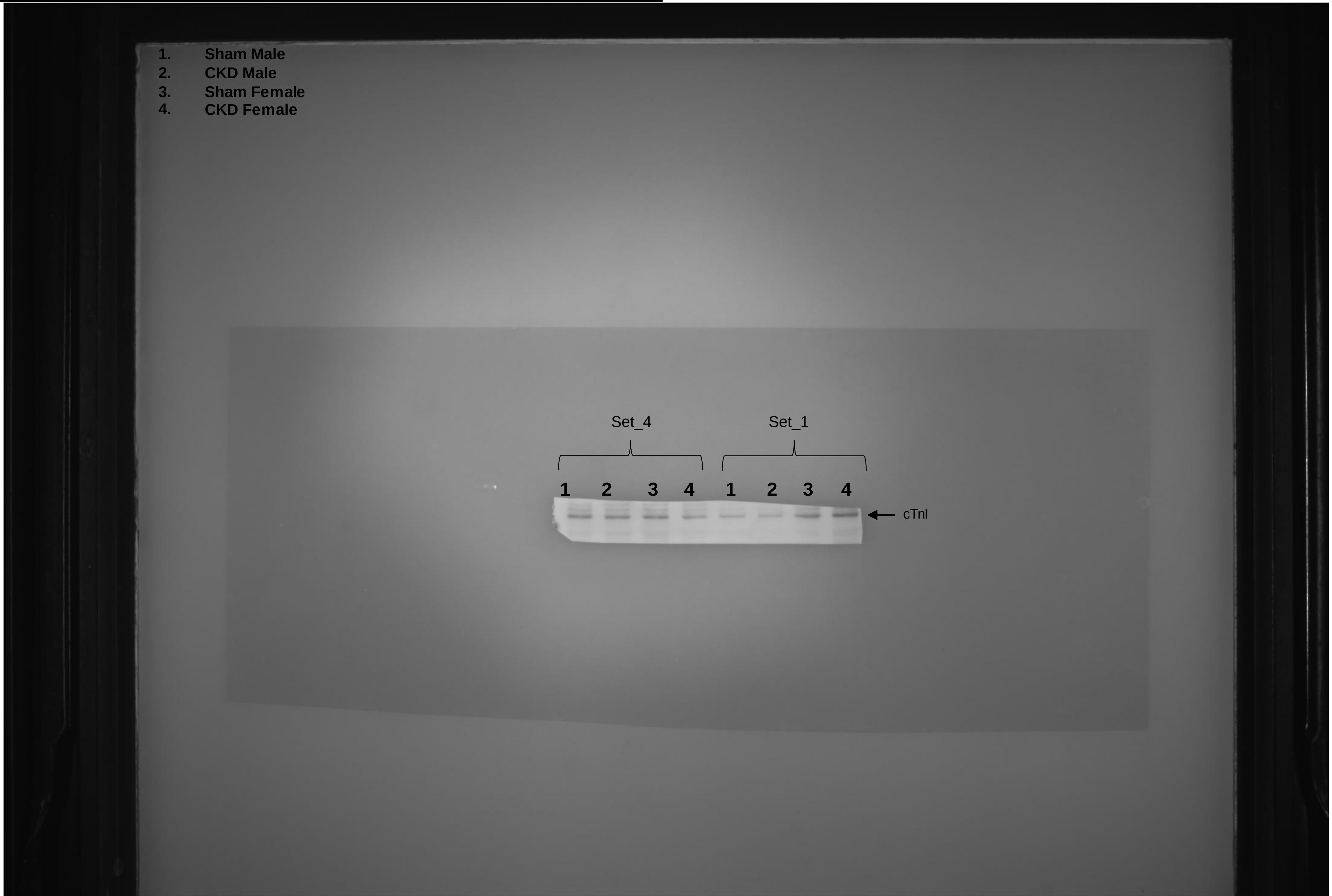

Left ventricle_Cardiac Troponin I_original membranes_Thr-144-P

S7A

1. Sham Male
2. CKD Male
3. Sham Female
4. CKD Female

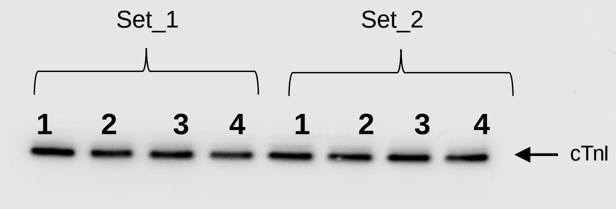

Left ventricle_Cardiac Troponin I_original membranes_ Thr-144-P-Blot stain

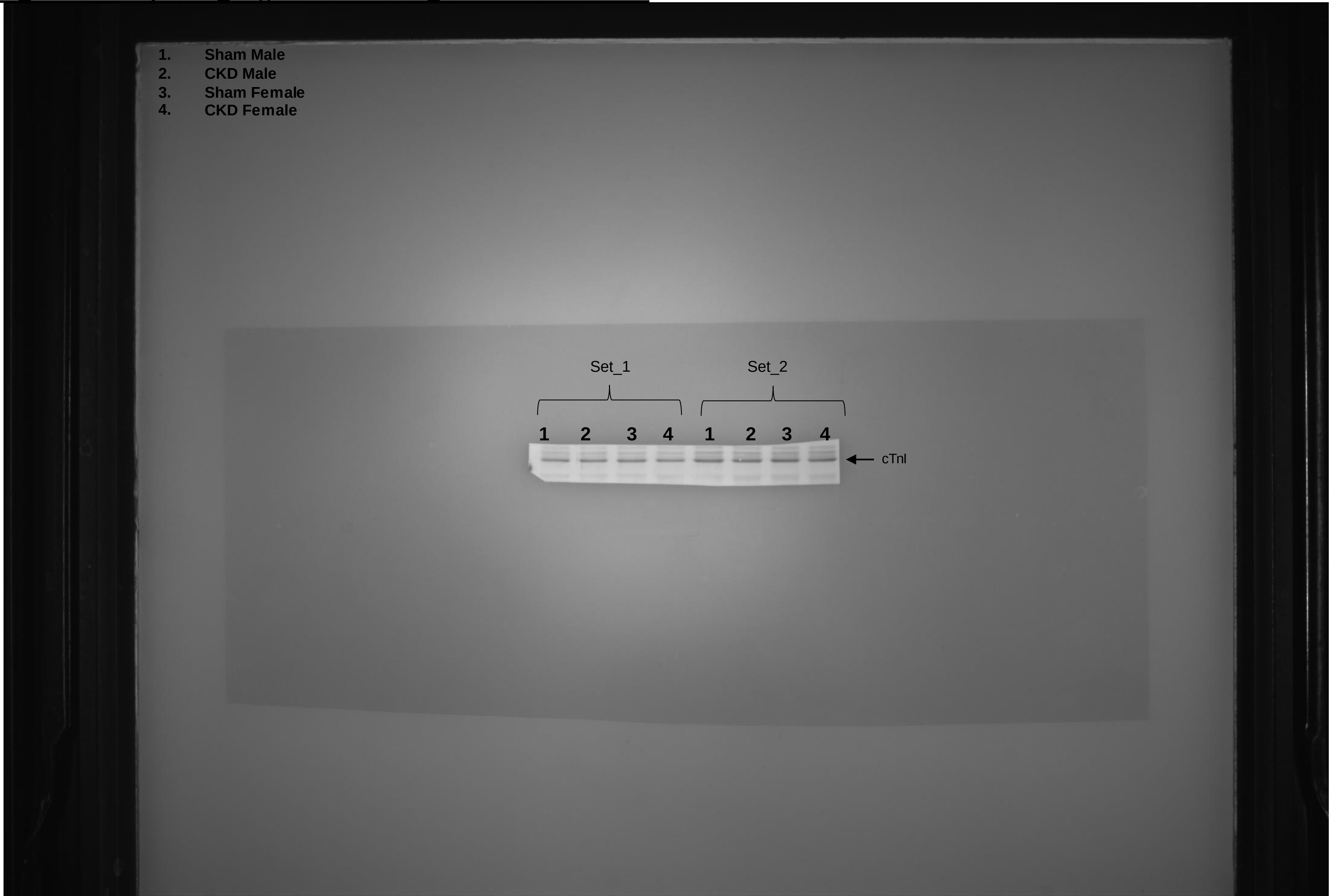

Left ventricle_Cardiac Troponin I_original membranes_Thr-144-P

S8A

1. Sham Male
2. CKD Male
3. Sham Female
4. CKD Female

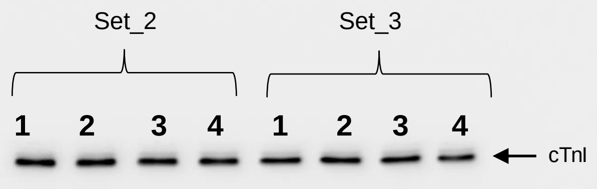

Left ventricle_Cardiac Troponin I_original membranes_ Thr-144-P-Blot stain

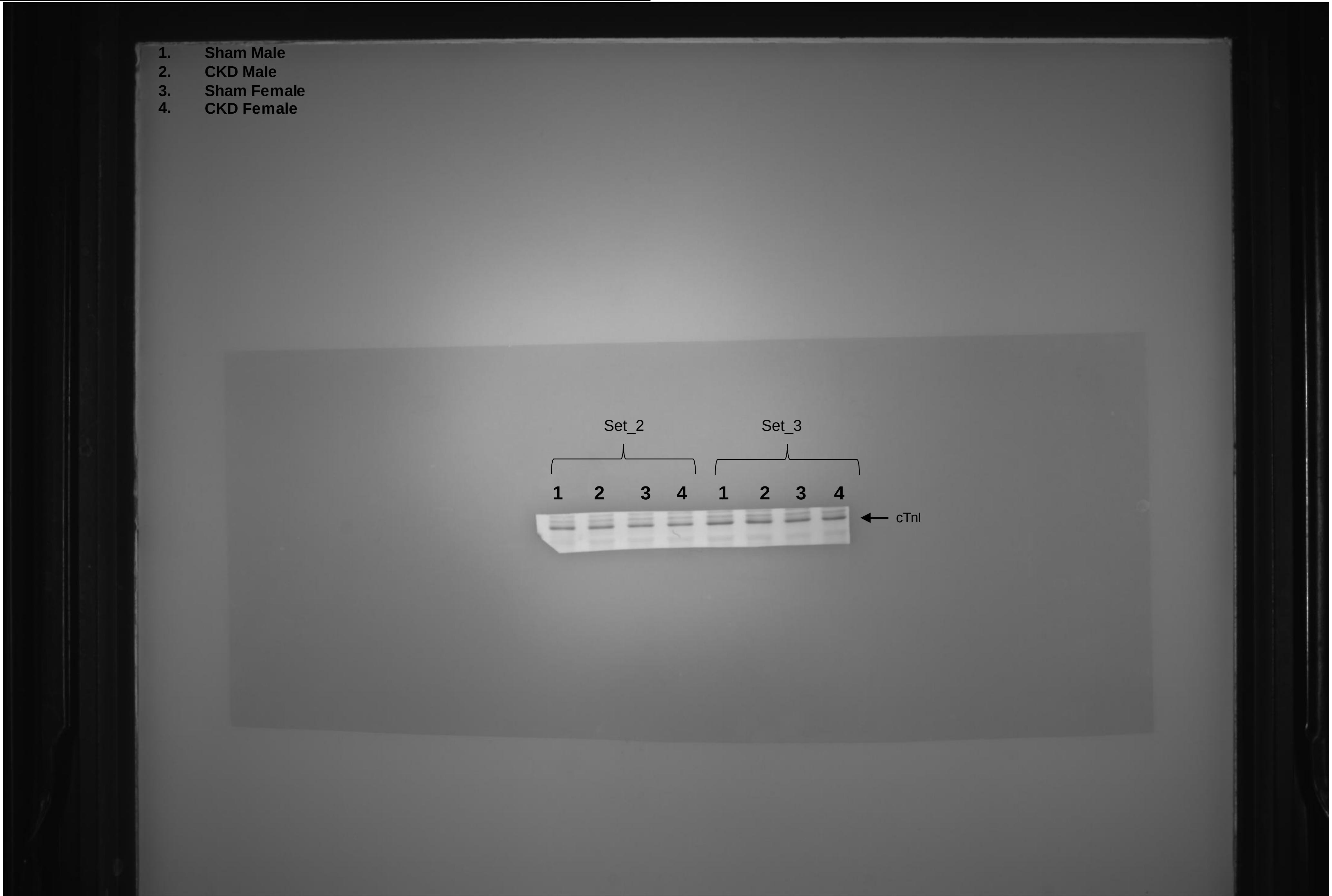

Left ventricle_Cardiac Troponin I_original membranes_Thr-144-P

S9A

1. Sham Male
2. CKD Male
3. Sham Female
4. CKD Female

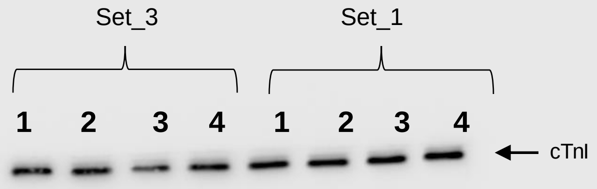

Left ventricle Cardiac Troponin I original membranes Thr-144-P-Blot stain

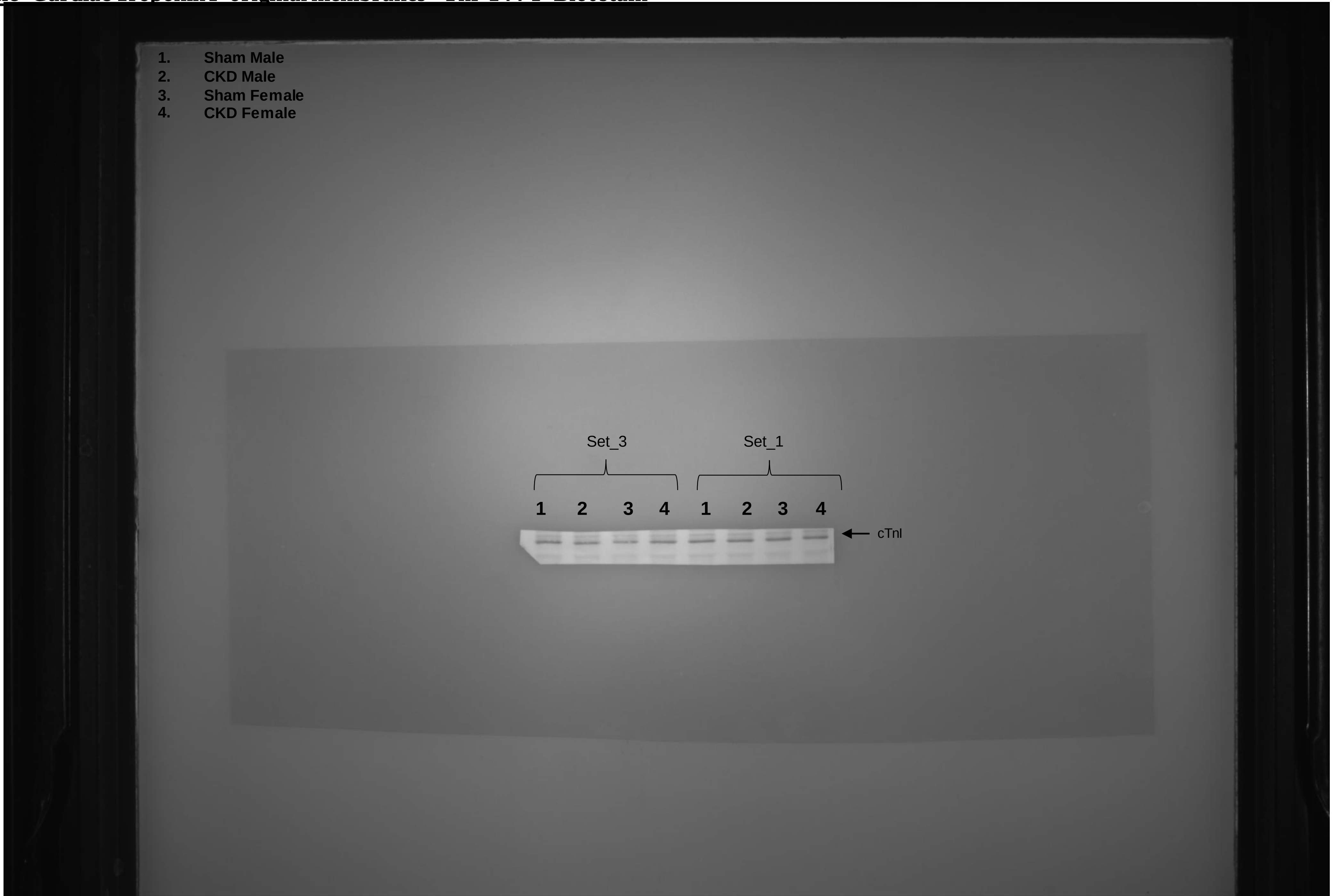

Left ventricle_Cardiac Troponin I_original membranes_Thr-144-P

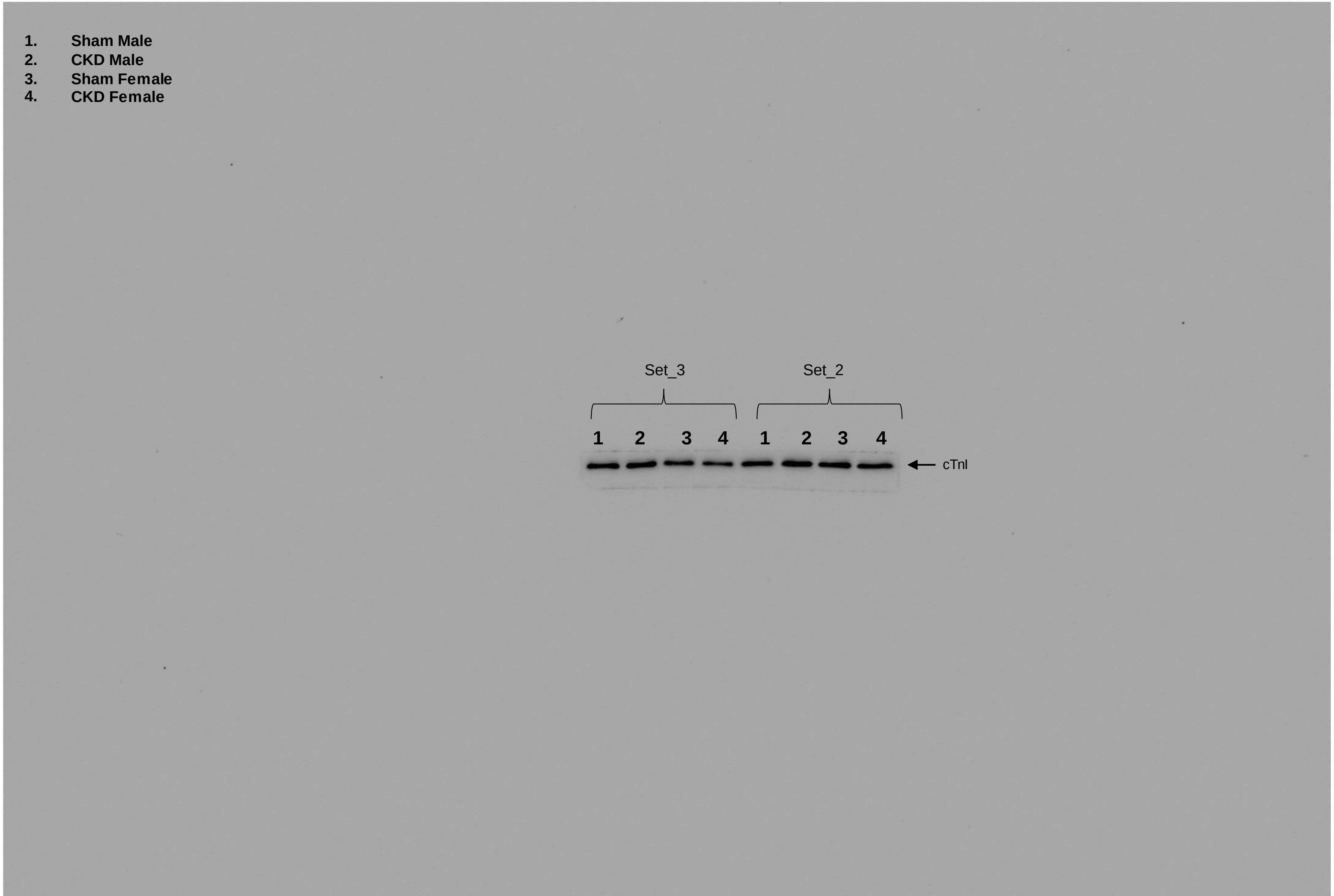

Left ventricle_Cardiac Troponin I_original membranes_ Thr-144-P-Blot stain

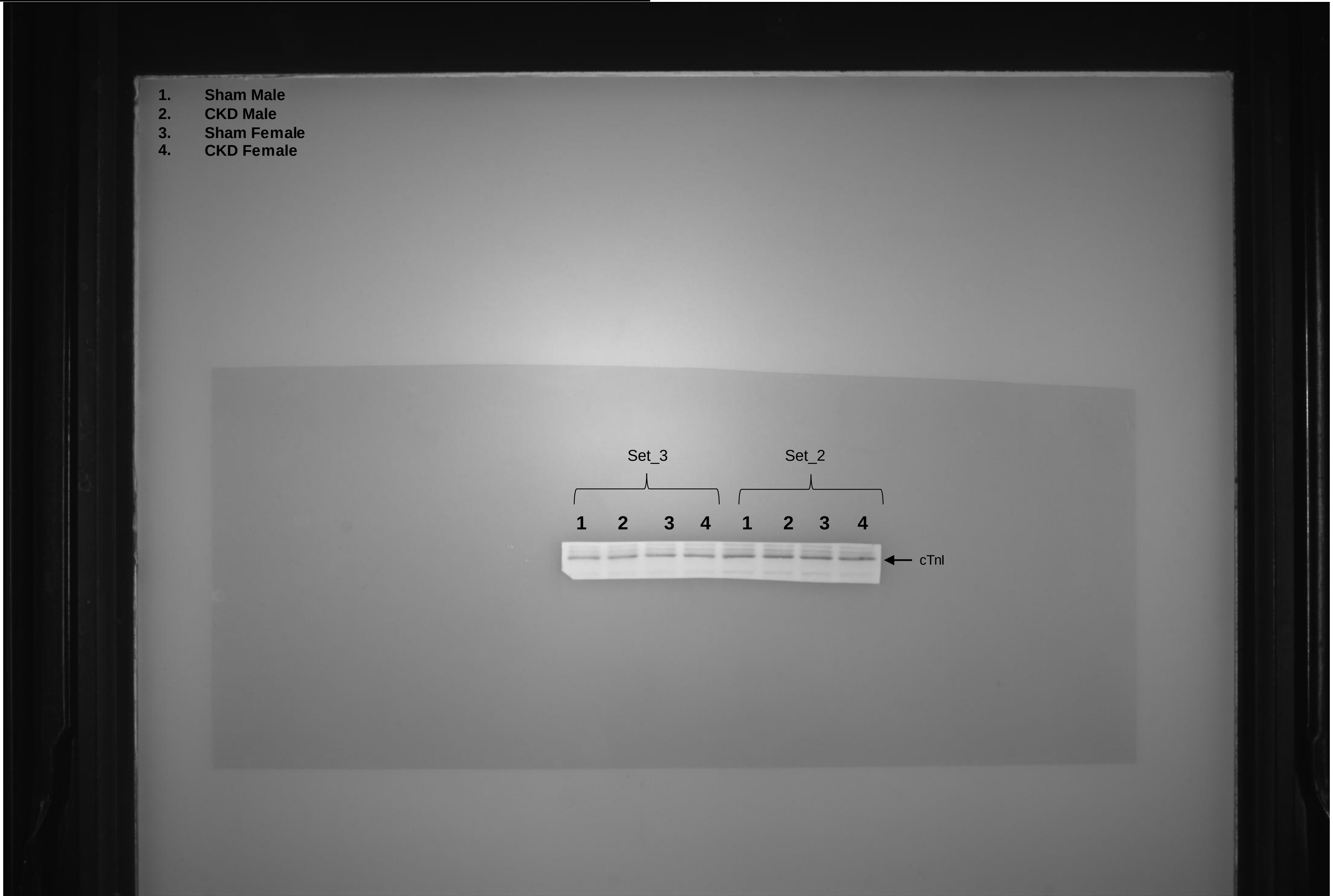

Left ventricle_Cardiac Troponin I_original membranes_Thr-144-P

S11A

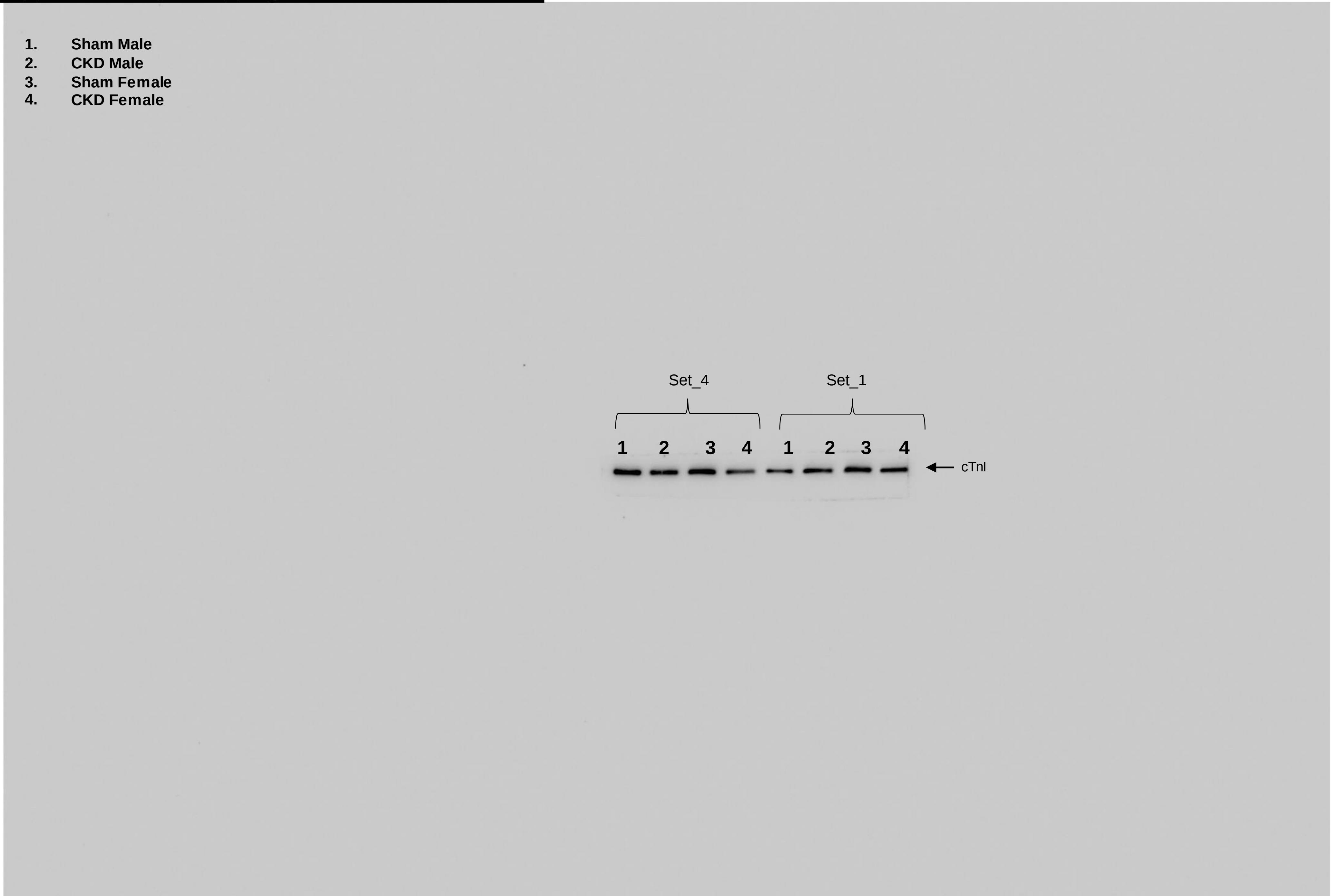

Left ventricle_Cardiac Troponin I_original membranes_ Thr-144-P-Blot stain

S11B

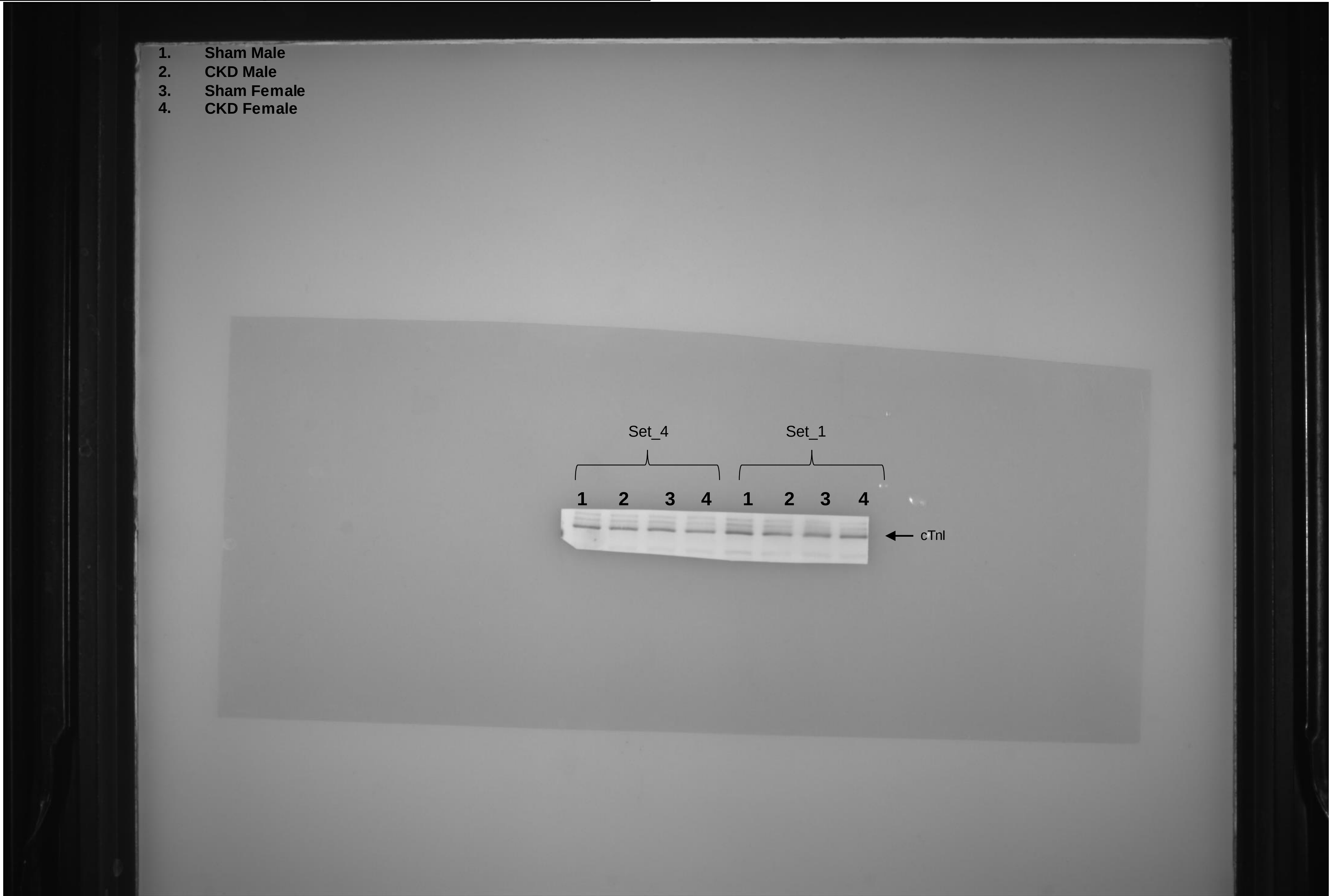

Left ventricle_Cardiac Troponin I_original membranes_Thr-144-P

S12A

1. Sham Male
2. CKD Male
3. Sham Female
4. CKD Female

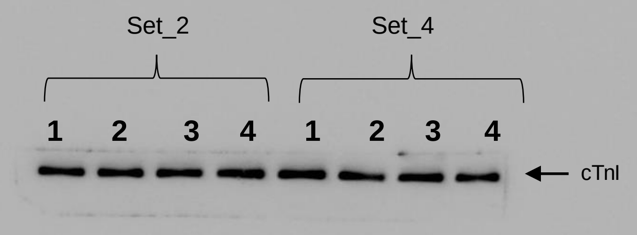

Left ventricle_Cardiac Troponin I_original membranes_ Thr-144-P-Blot stain

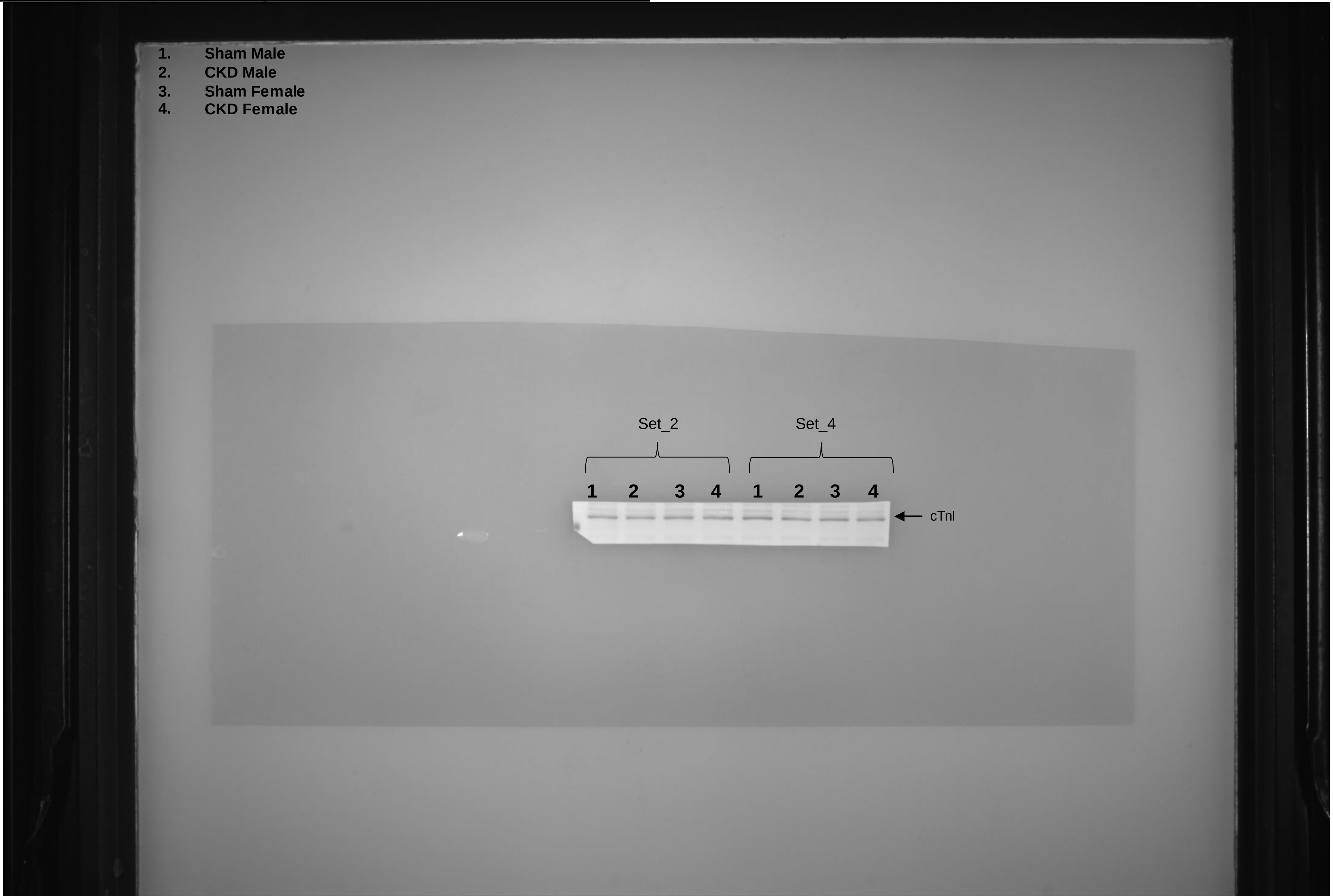

Left ventricle_Cardiac Troponin I_original membranes_Ser-43-P

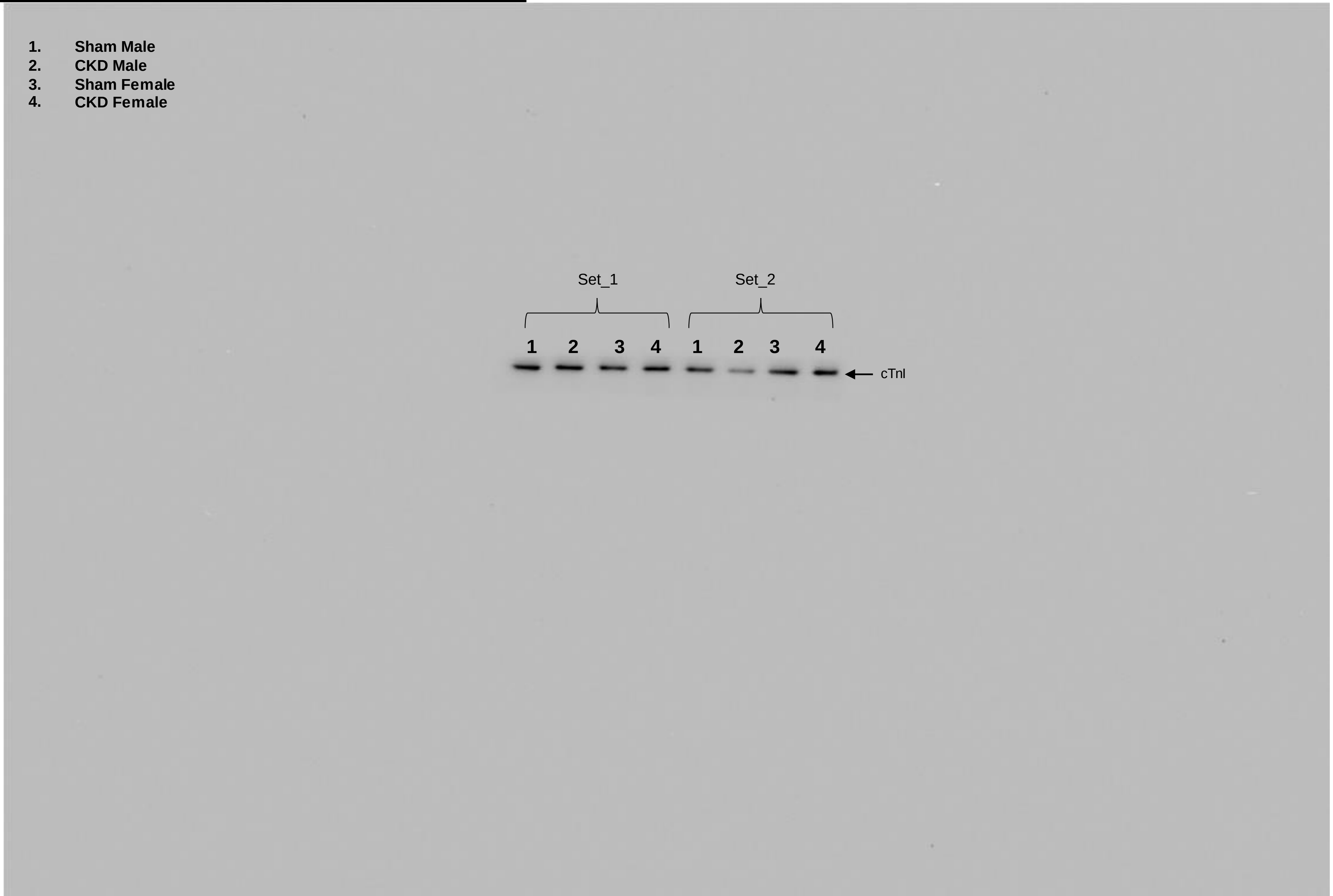

Left ventricle_Cardiac Troponin I_original membranes_Ser-43-P-Blot stain

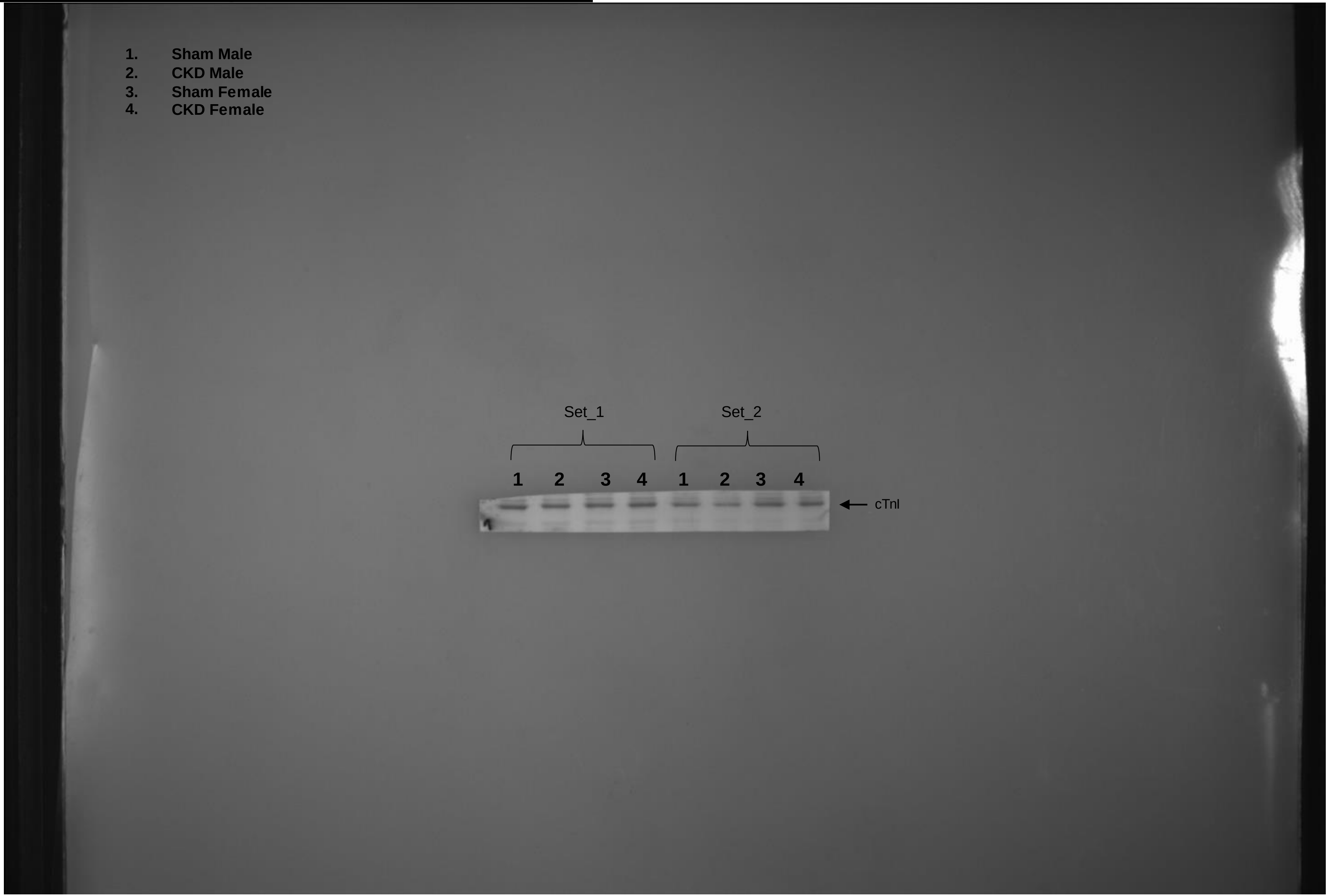

S14A

Left ventricle_Cardiac Troponin I_original membranes_Ser-43-P

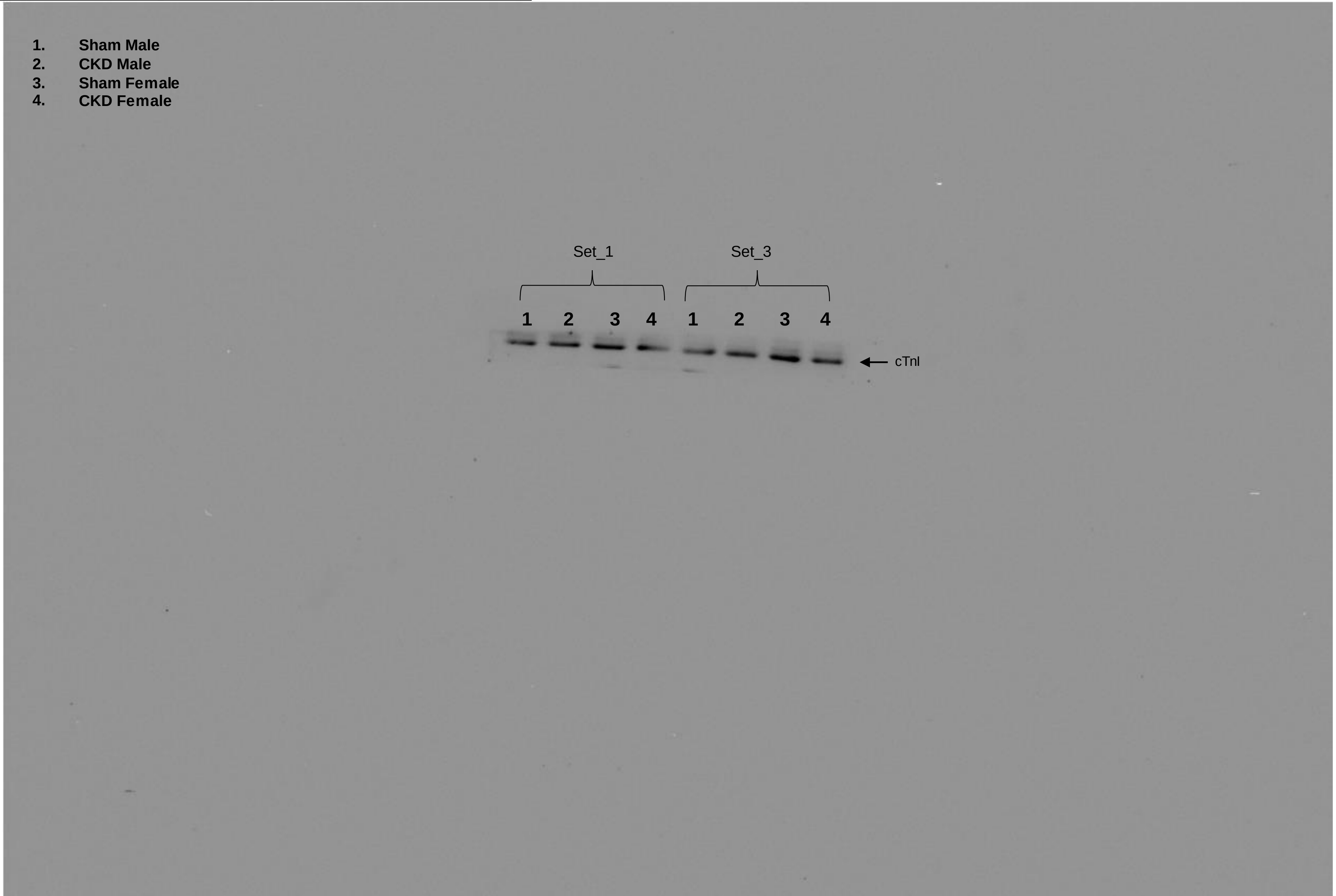

Left ventricle_Cardiac Troponin I_original membranes_Ser-43-P-Blot stain

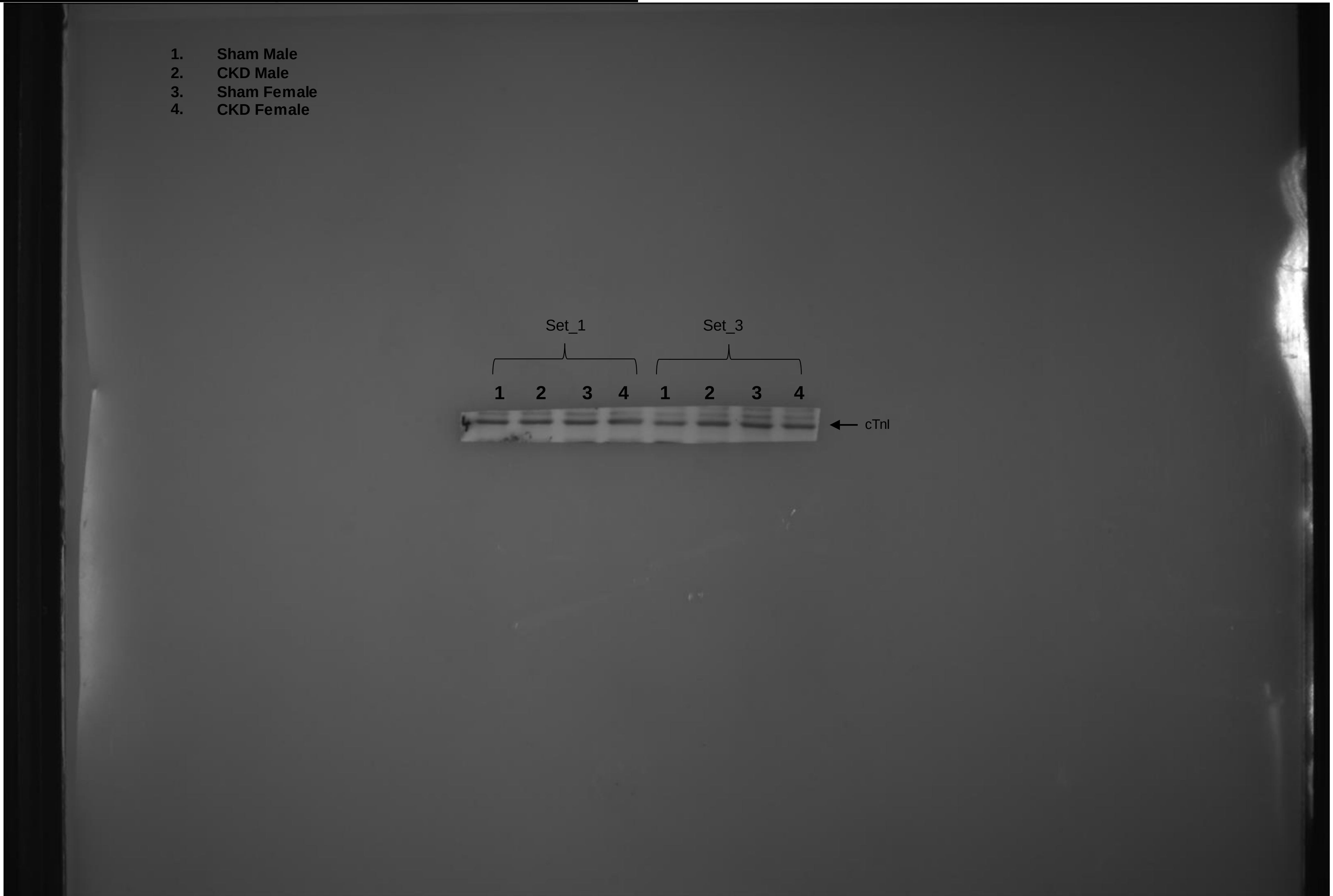

Left ventricle_Cardiac Troponin I_original membranes_Ser-43-P

S15A

1. Sham Male
2. CKD Male
3. Sham Female
4. CKD Female

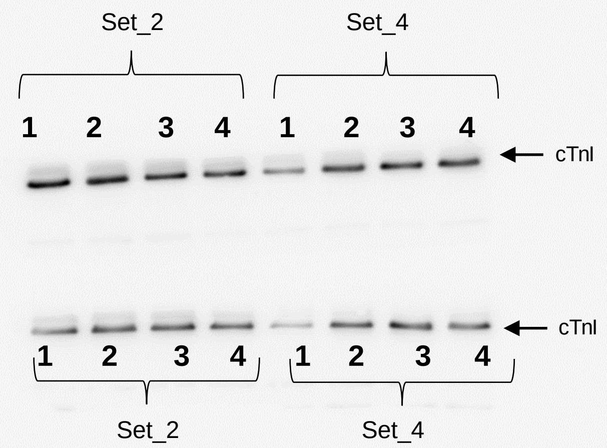

Left ventricle_Cardiac Troponin I_original membranes_Ser-43-P-Blot stain

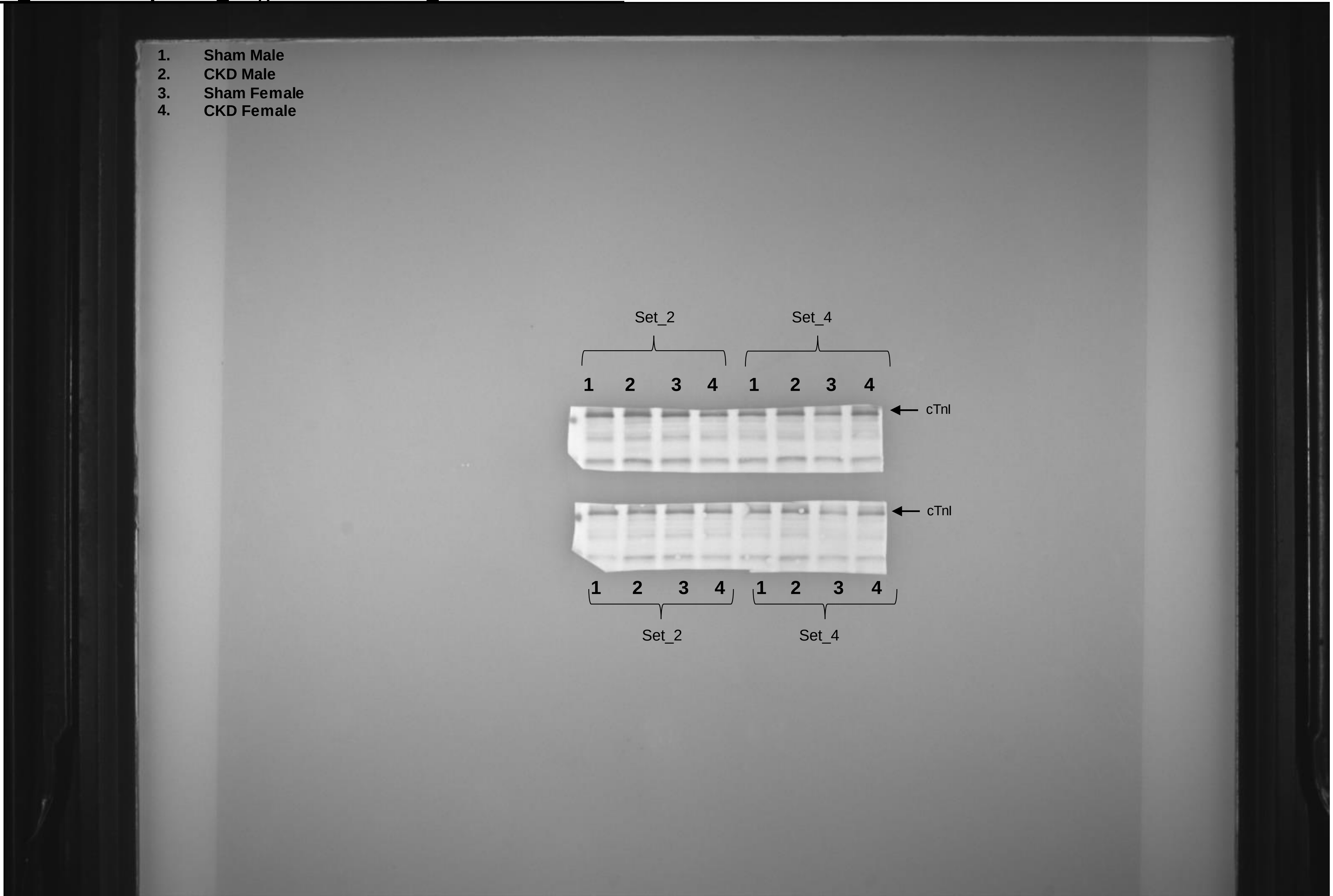

Left ventricle_Cardiac Troponin I_original membranes_Ser-43-P

S16A

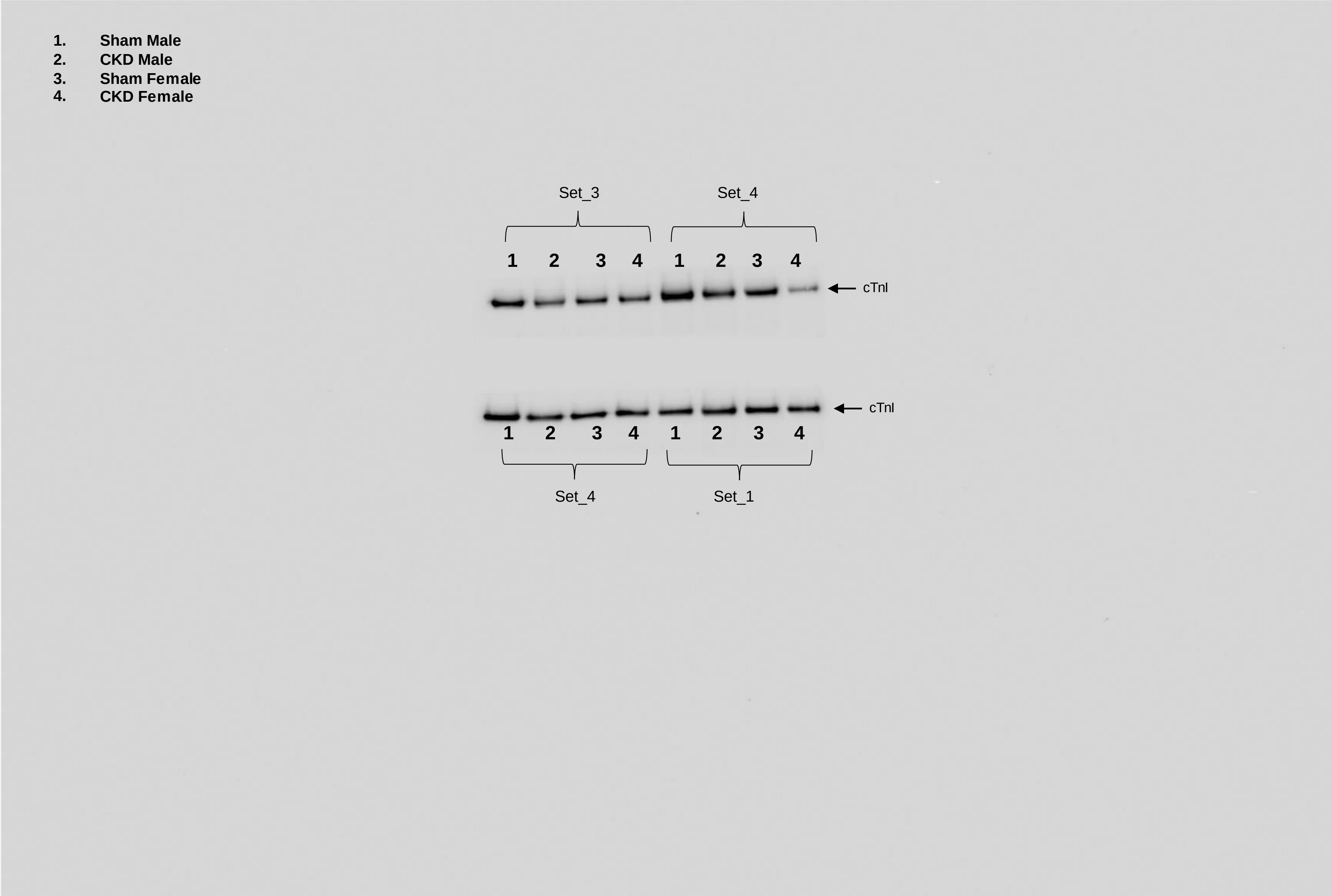

Left ventricle_Cardiac Troponin I_original membranes_Ser-43-P-Blot stain

S16B

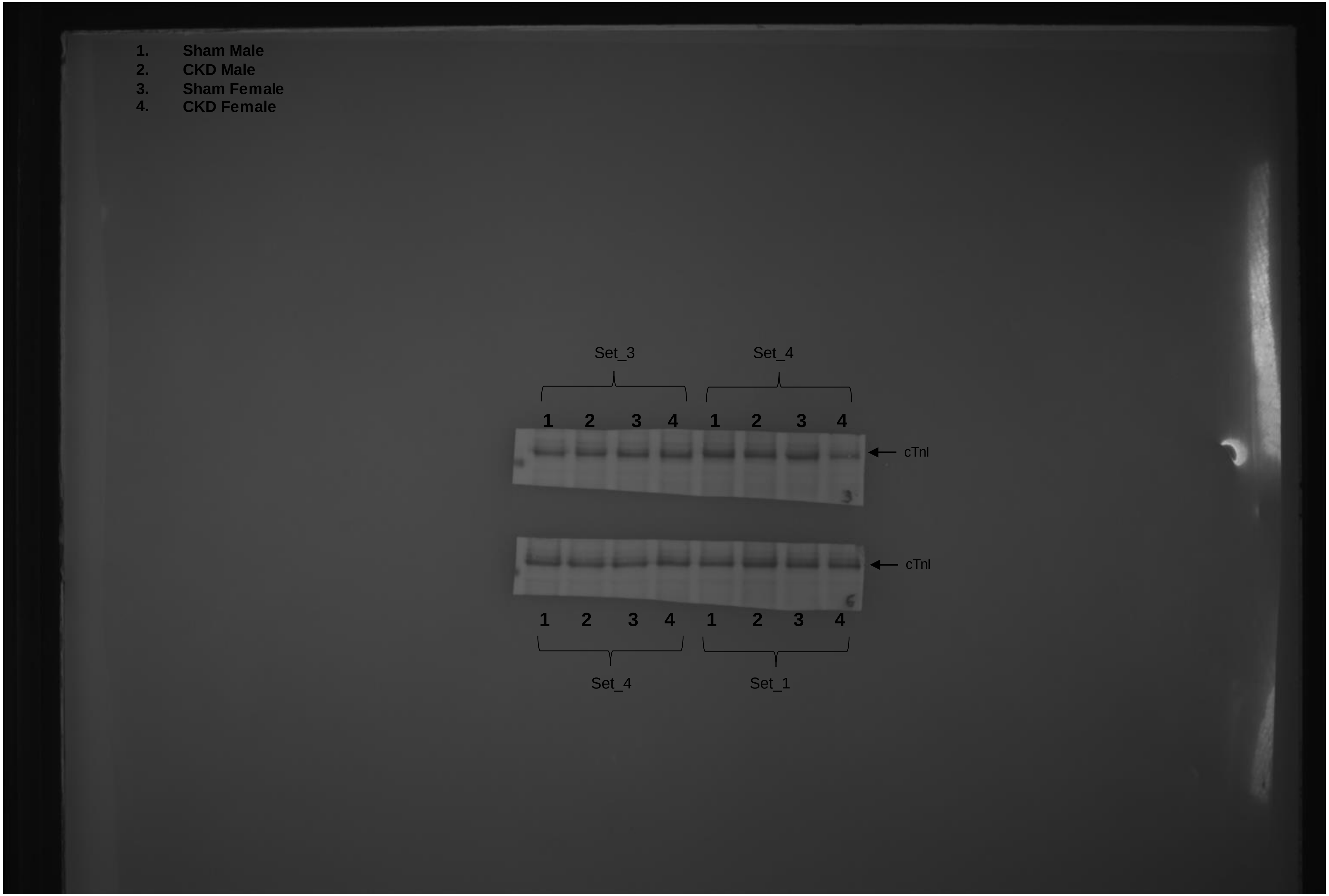

Left ventricle_Cardiac myosin binding protein C_original membranes_ Ser-282-P

S17A

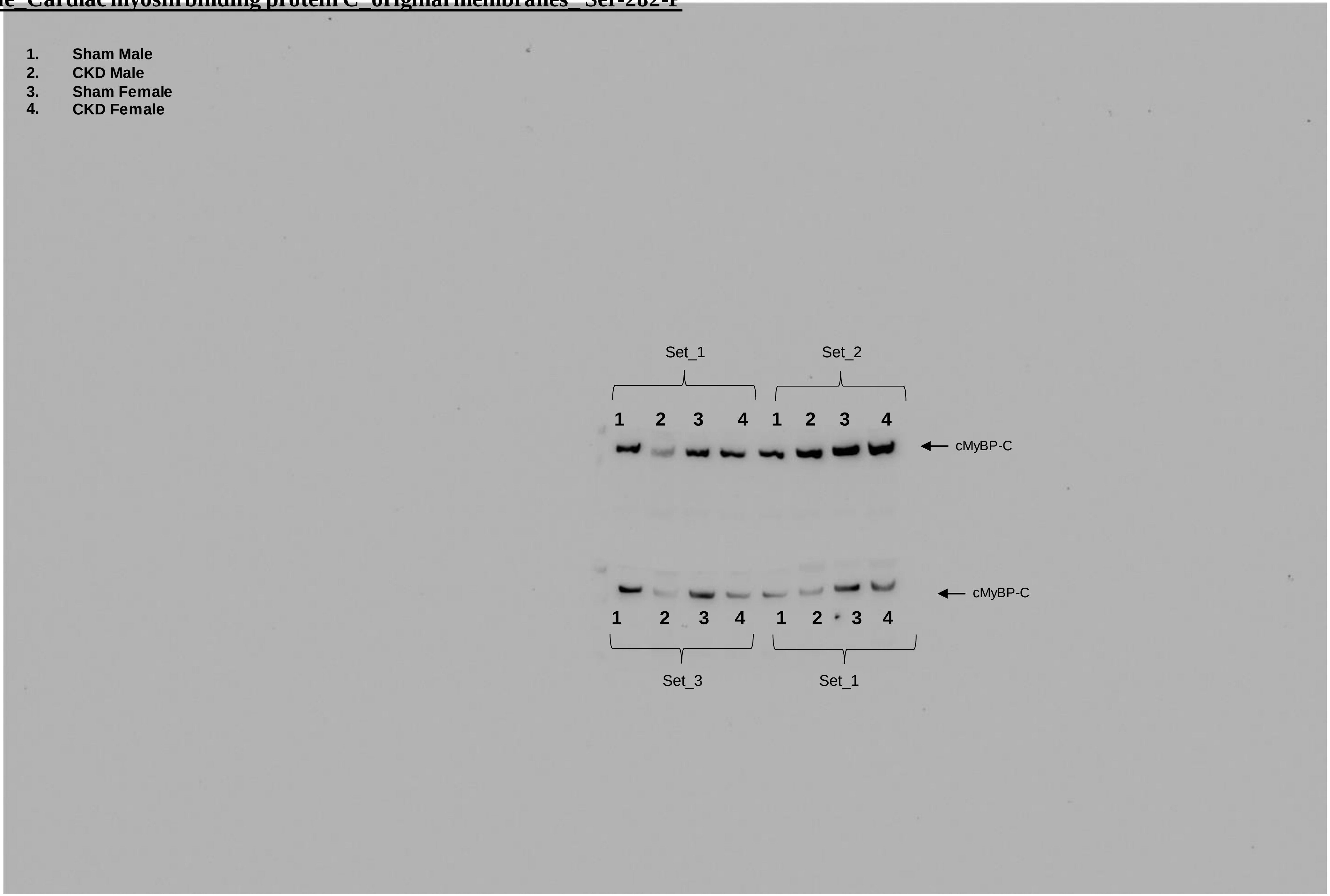

Left ventricle_Cardiac myosin binding protein C_original membranes_ Ser-282-P-Blot stain

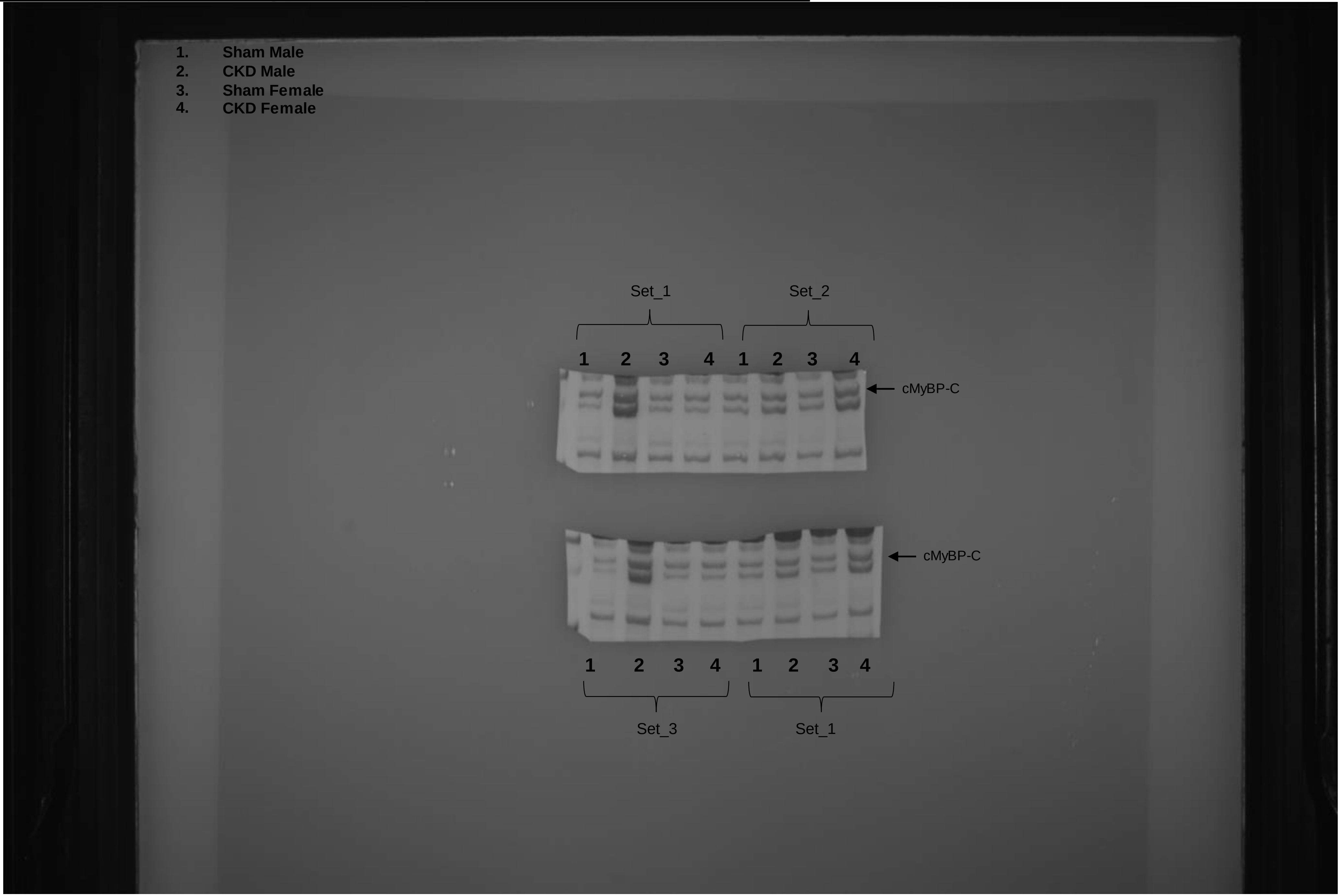

Left ventricle_Cardiac myosin binding protein C_original membranes_ Ser-282-P

S18A

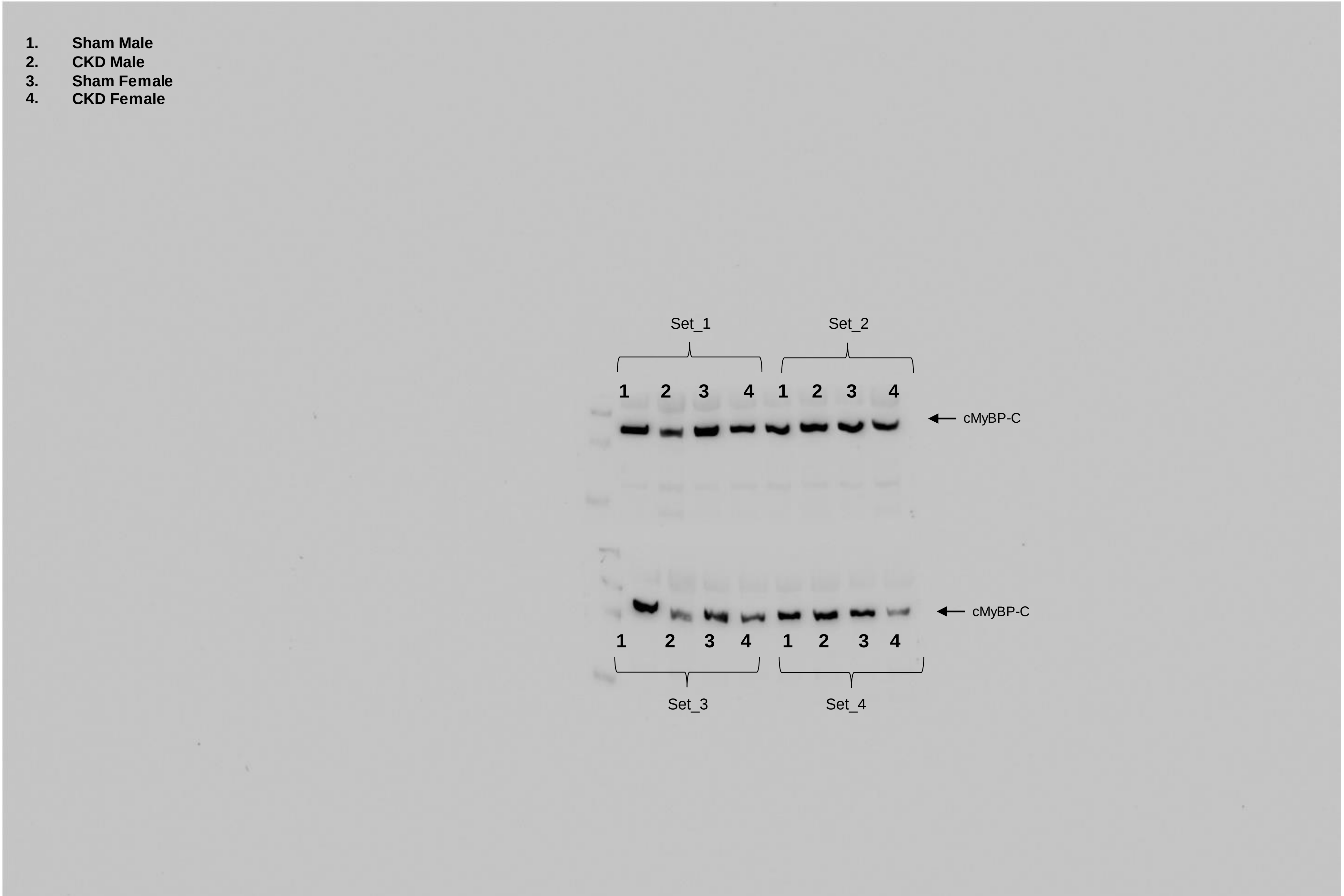

Left ventricle_Cardiac myosin binding protein C_original membranes_ Ser-282-P-Blot stain

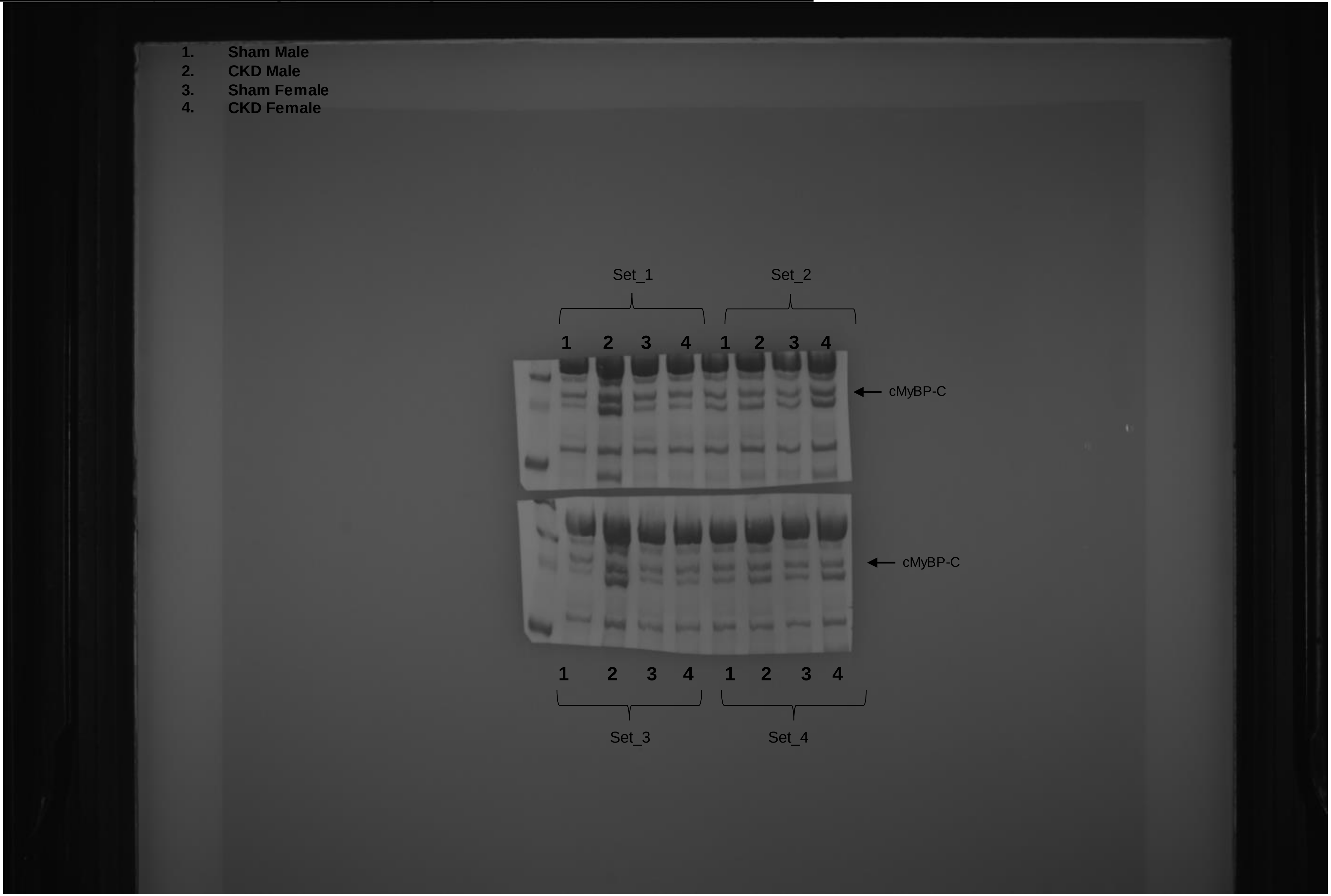

Left ventricle_Cardiac myosin binding protein C_original membranes_ Ser-282-P

1. **Sham Male**
2. **CKD Male**
3. **Sham Female**
4. **CKD Female**

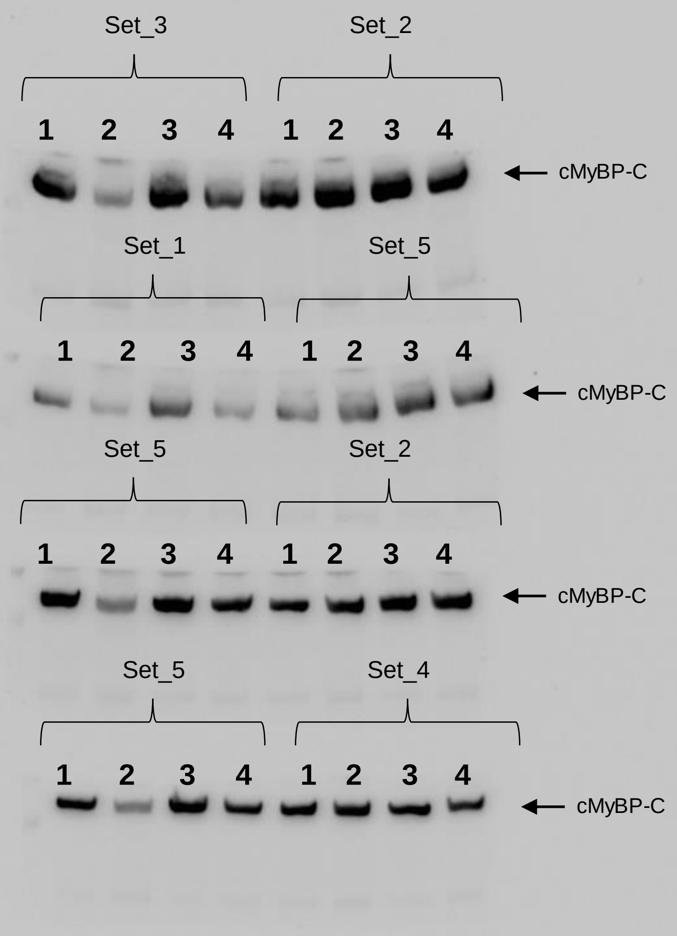

Left ventricle_Cardiac myosin binding protein C_original membranes_ Ser-282-P-Blot stain

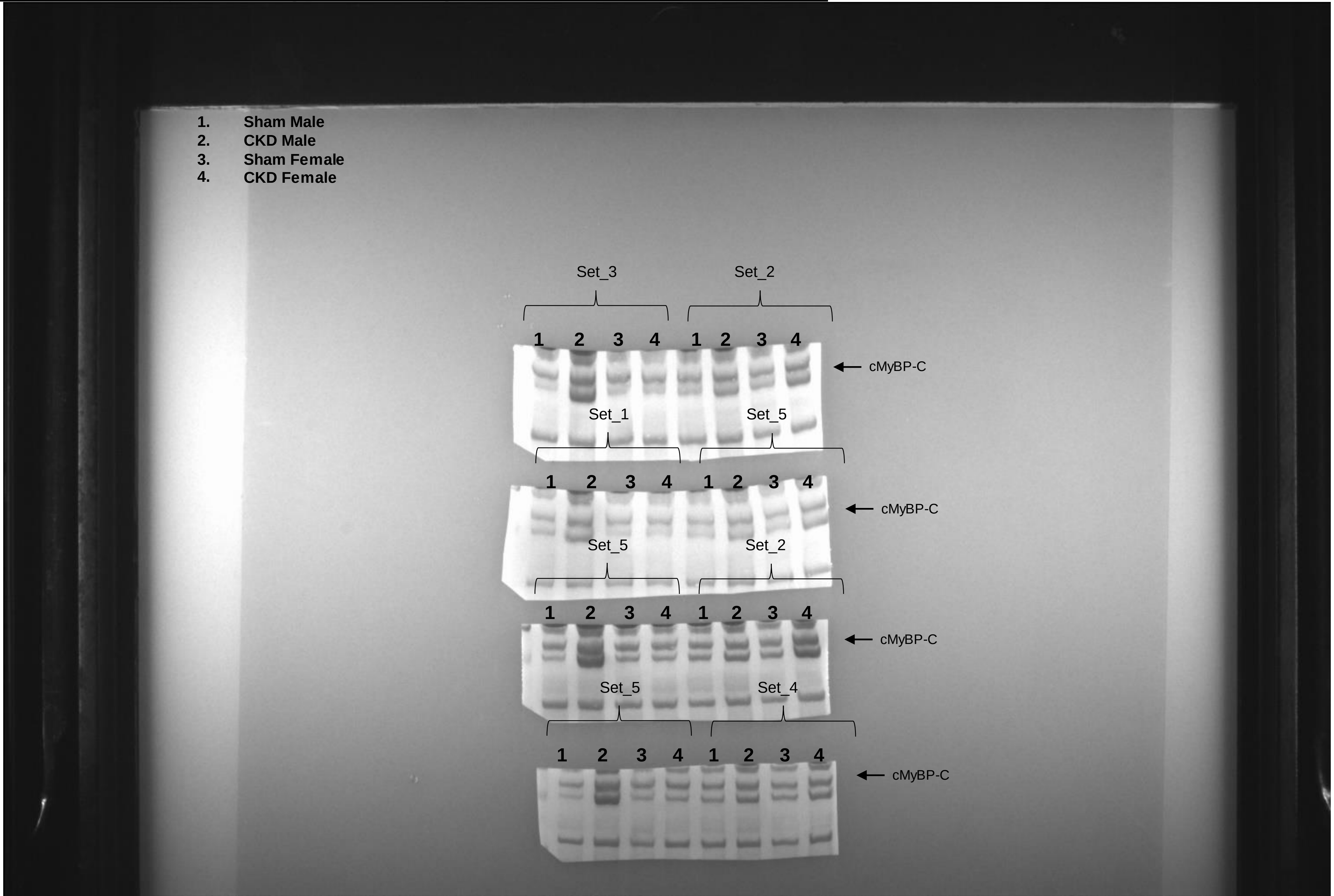

Left ventricle_Titin-P_original gel

S20A

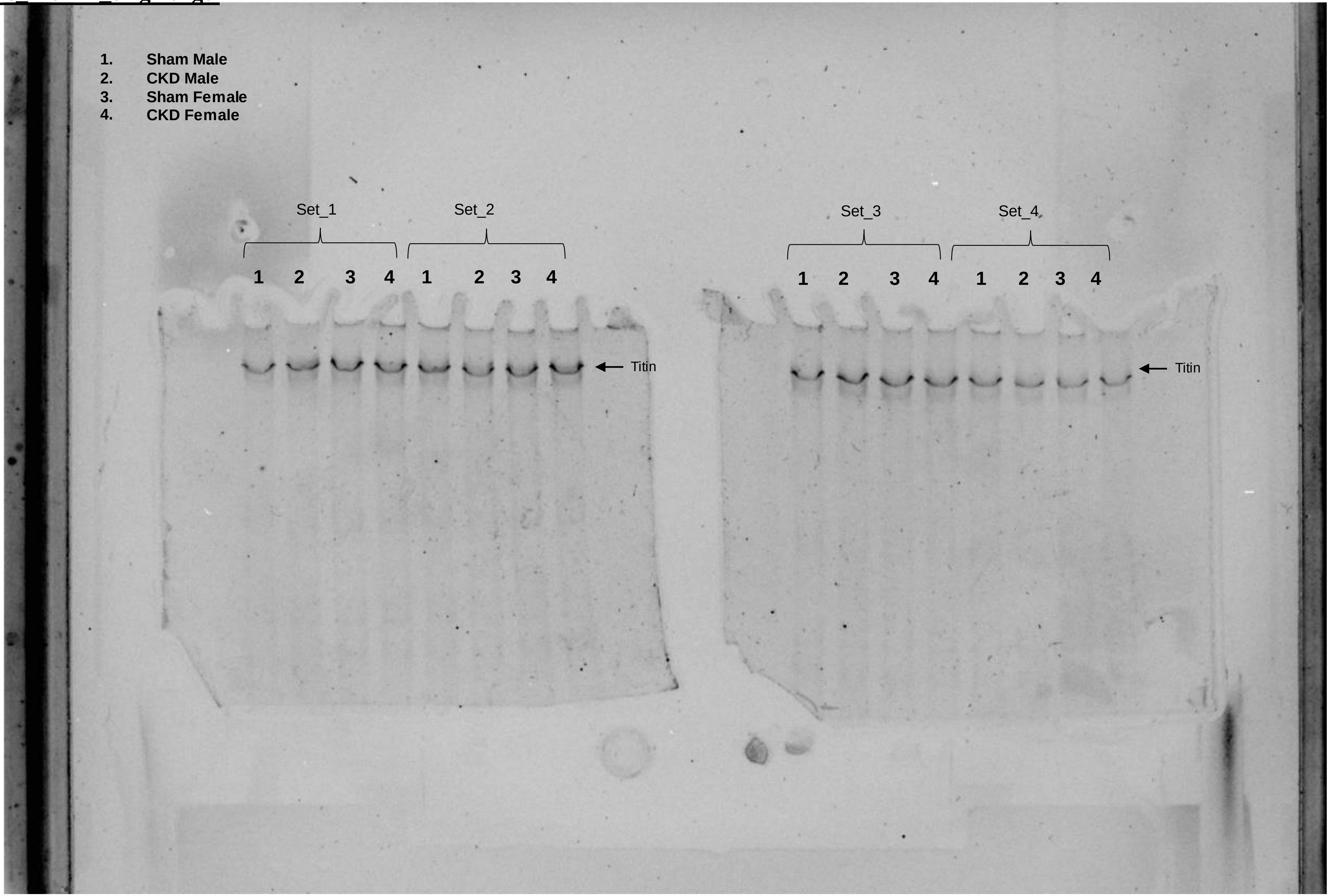

Left ventricle_Titin-total protein_original gel

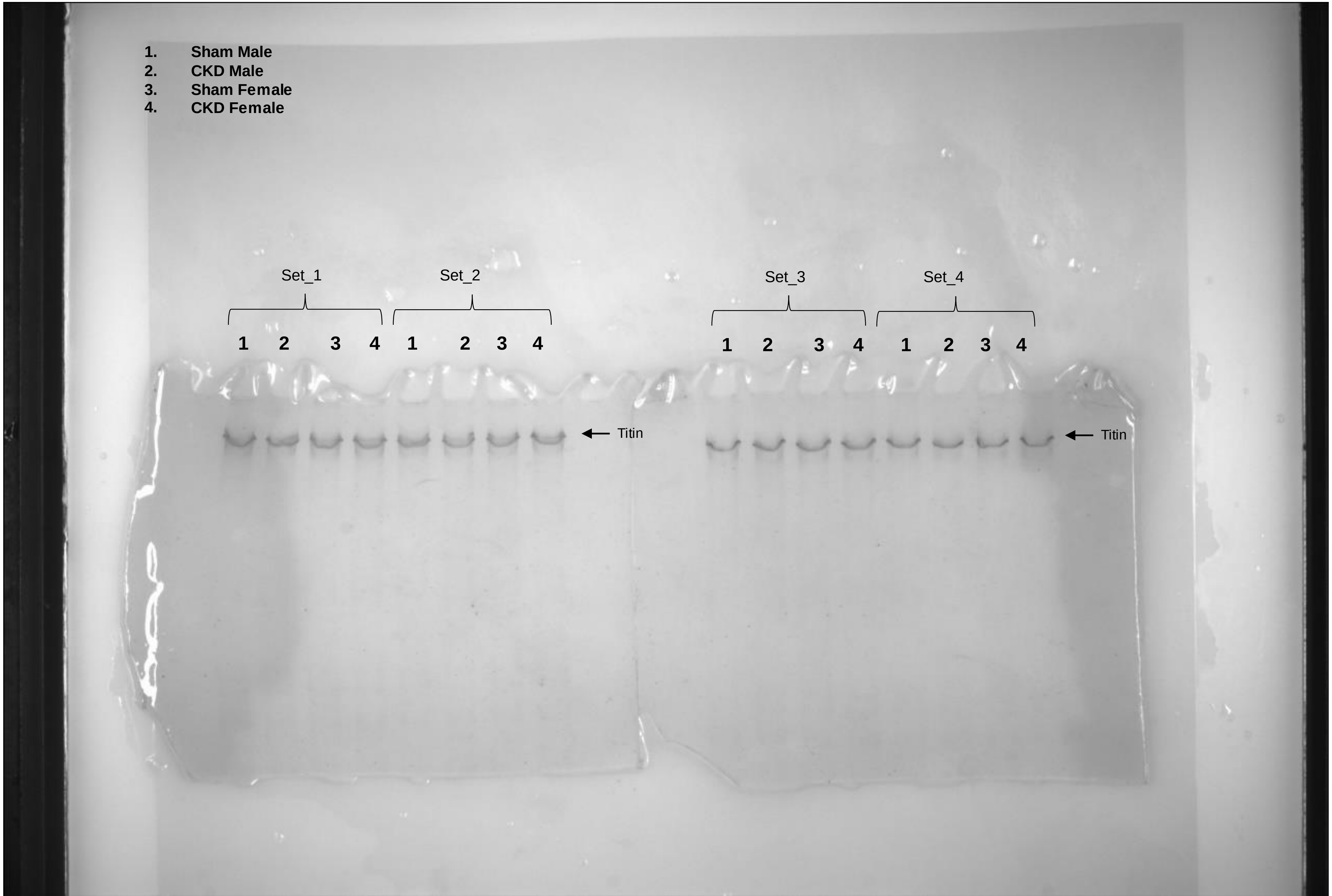

Left ventricle_Titin-P_original gel

S21A

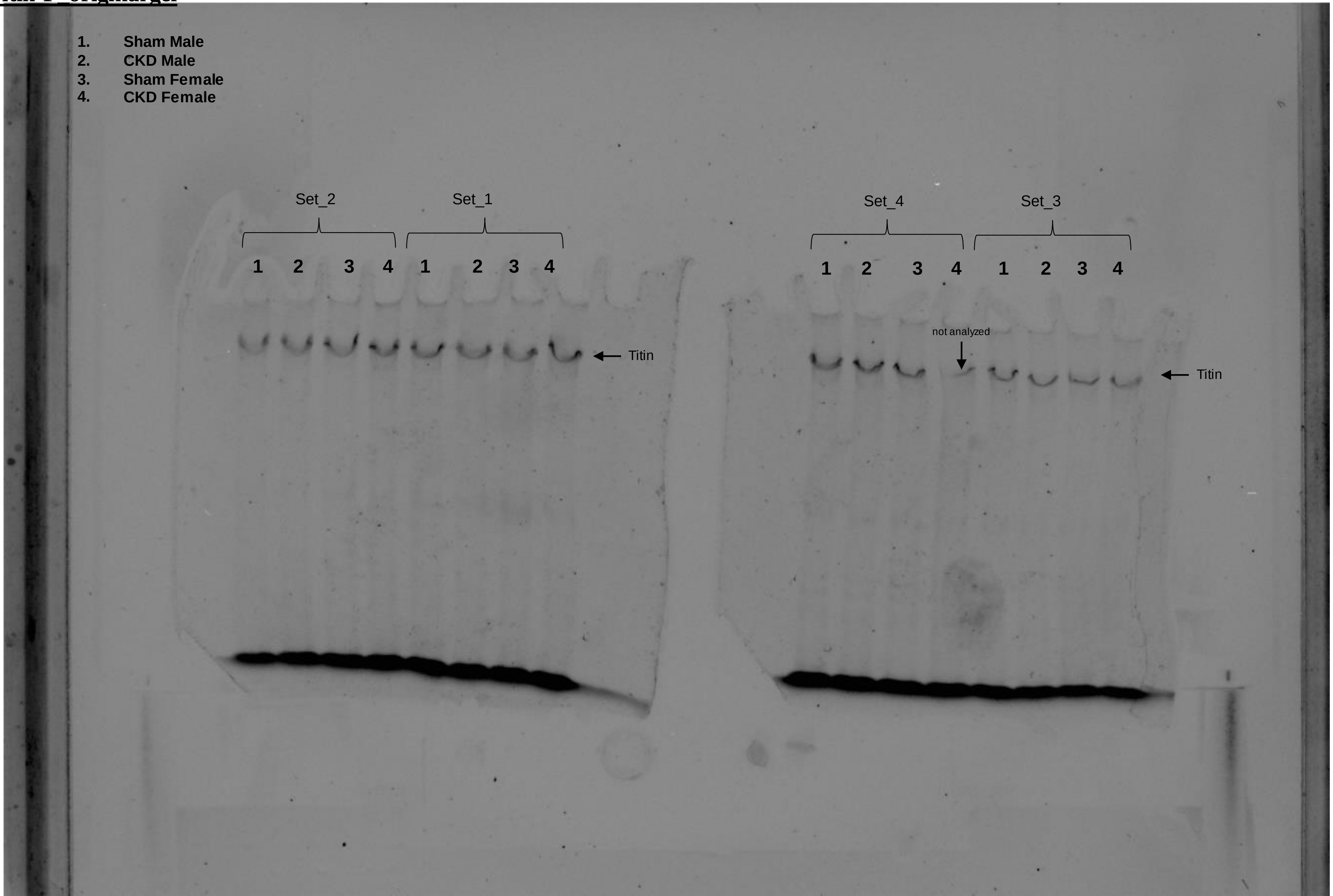

Left ventricle_Titin-total protein_original gel

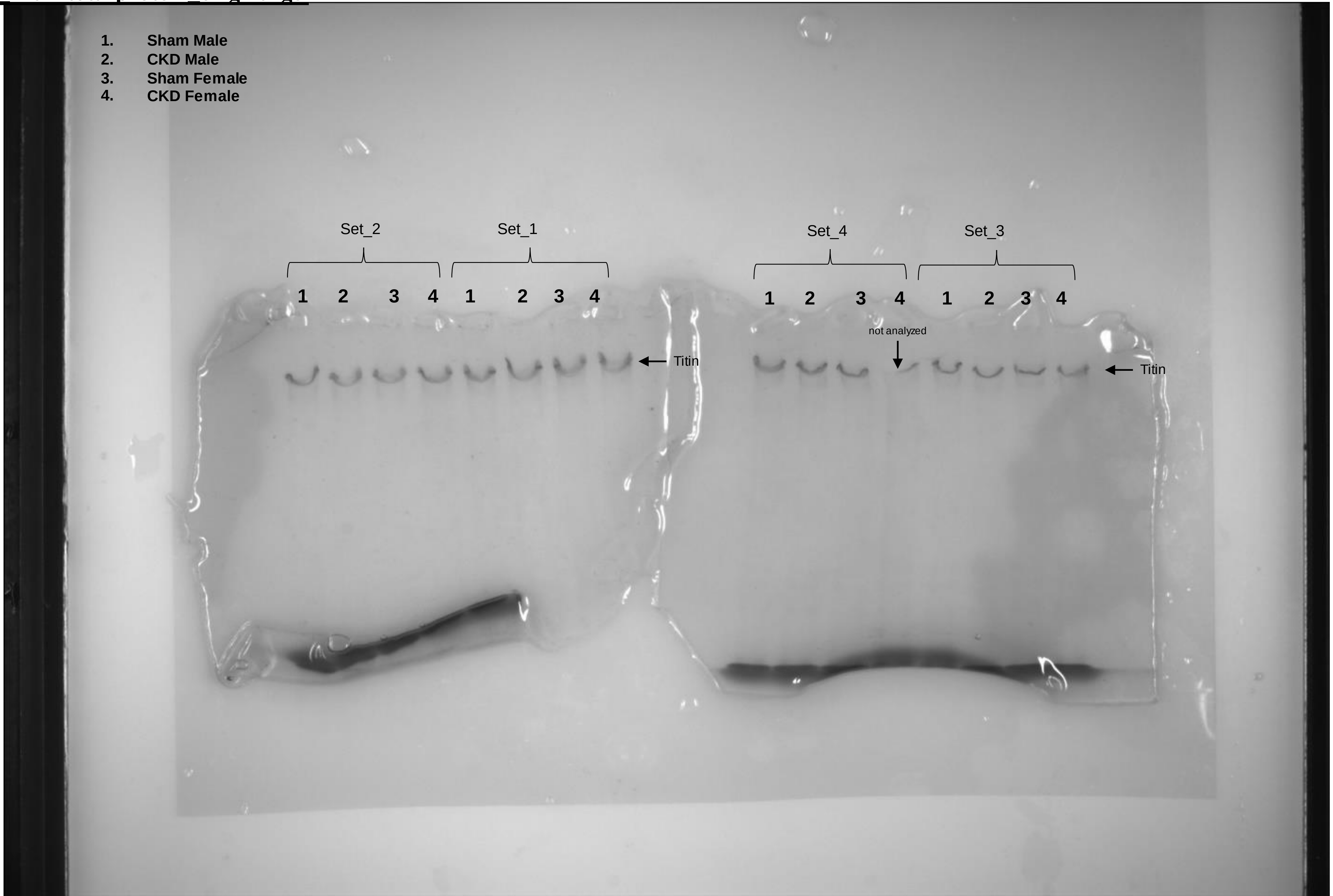

Left ventricle_Titin-P_original gel

S22A

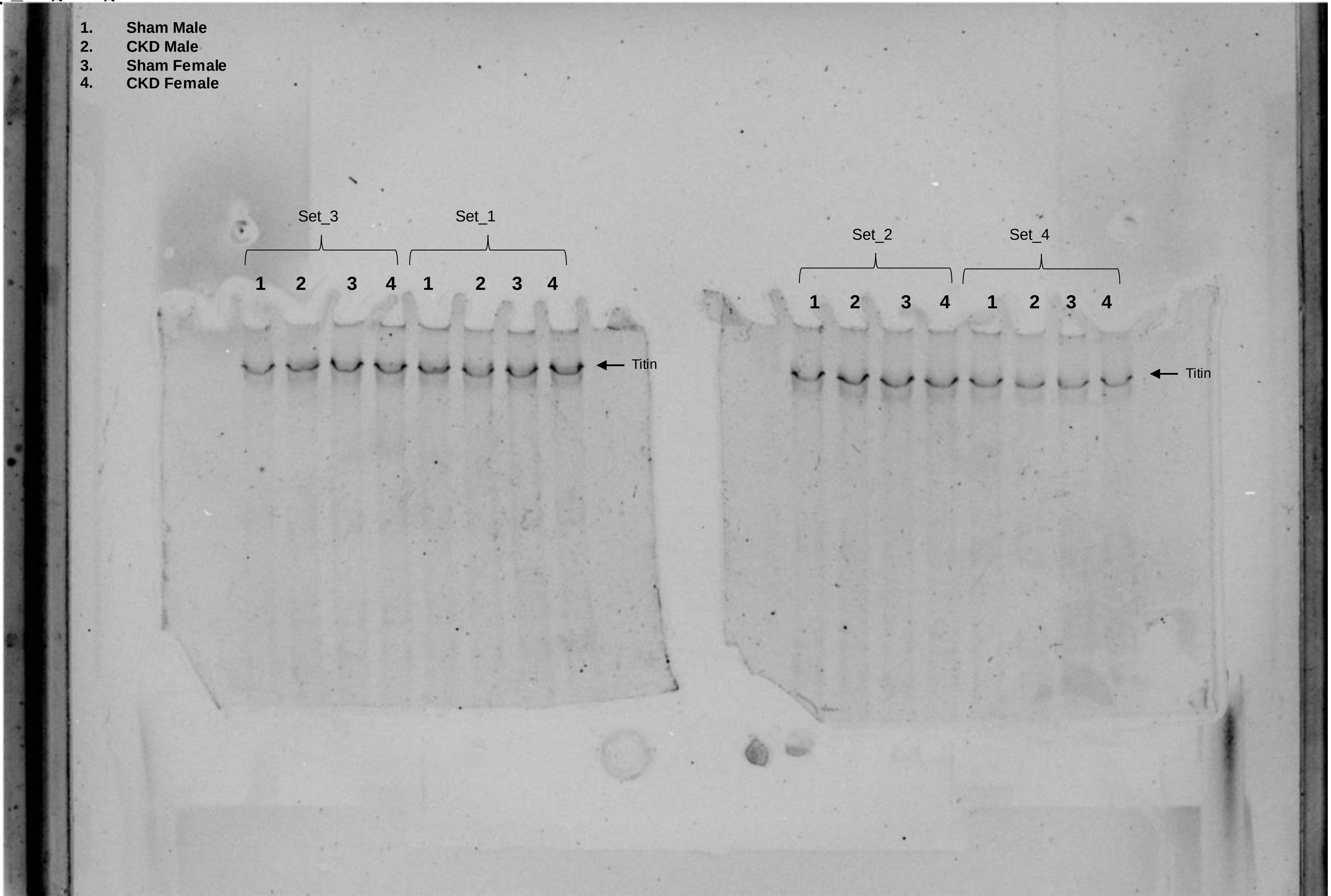

Left ventricle_Titin-total protein_original gel

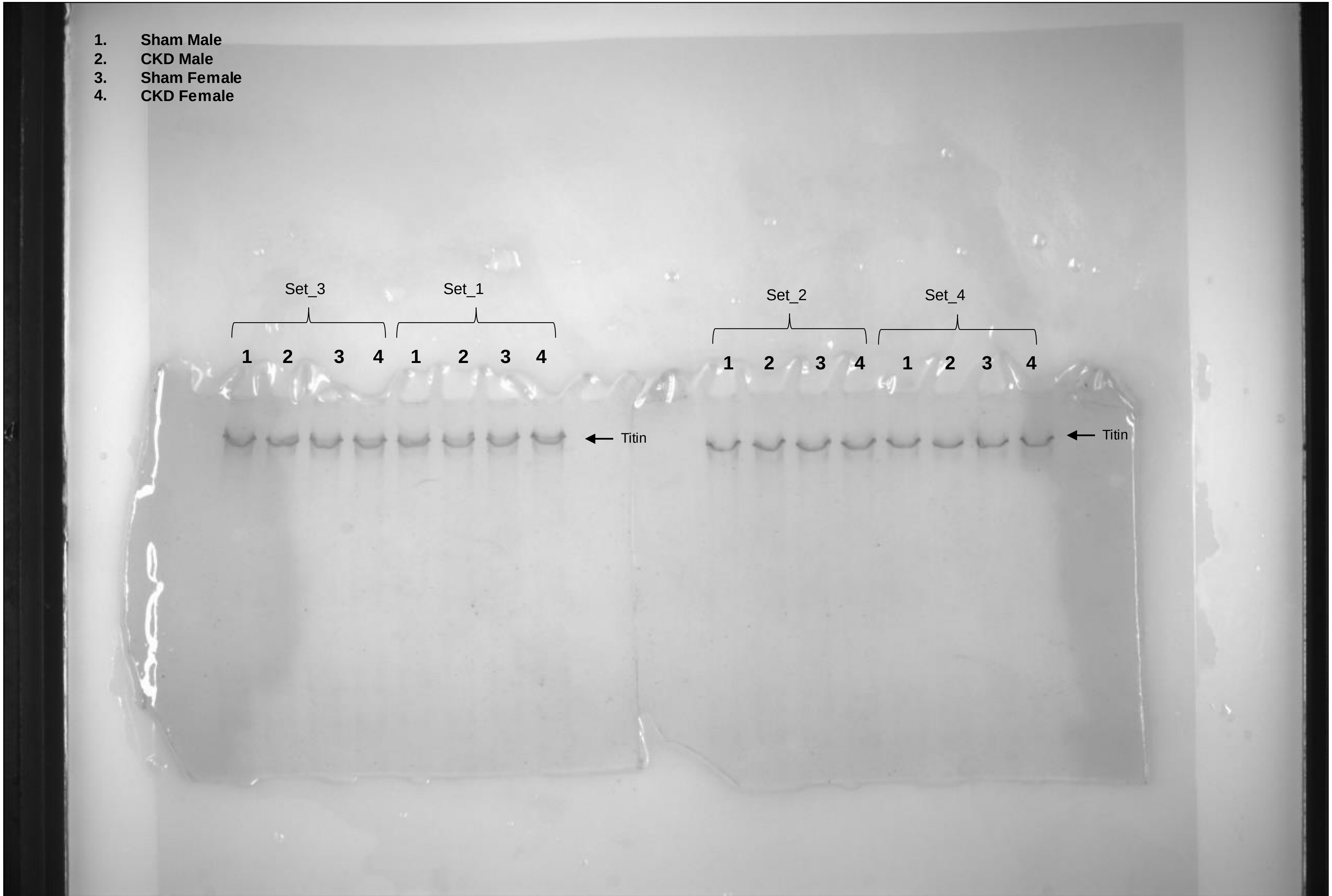

Supplement: Supplementary file 1 [file ijms-26-02259-s001.zip › ijms-3470141-supplementary.pdf]
